# The attenuation effect of potassium 2‐(1‐hydroxypentyl)‐benzoate in a mouse model of diabetes‐associated cognitive decline: The protein expression in the brain

**DOI:** 10.1111/cns.13847

**Published:** 2022-04-20

**Authors:** Wenwen Yu, Huajing Yin, Yingni Sun, Si Shi, Jiang Li, Xiaoliang Wang

**Affiliations:** ^1^ State Key Laboratory of Bioactive Substances and Functions of Natural Medicines Department of Pharmacology Institute of materia Medica Chinese Academy of Medical Sciences & Peking Union Medical College Beijing China

**Keywords:** 2D‐DIGE, Alzheimer's disease, diabetic encephalopathy, *dl*‐PHPB, proteomics, type 2 diabetes mellitus

## Abstract

**Aims:**

*dl*‐PHPB (potassium 2‐(1‐hydroxypentyl)‐benzoate) has been shown to have neuroprotective effects against acute cerebral ischemia, vascular dementia, and Alzheimer's disease. The aim of this study was to investigate the effects of *dl*‐PHPB on memory deficits and preliminarily explore the underlying molecular mechanism.

**Methods:**

Blood glucose and behavioral performance were evaluated in the KK‐A^y^ diabetic mouse model before and after *dl*‐PHPB administration. Two‐dimensional difference gel electrophoresis (2D‐DIGE)‐based proteomics was used to identify differentially expressed proteins in brain tissue. Western blotting was used to study the molecular mechanism of the related signaling pathways.

**Results:**

Three‐month‐old KK‐A^y^ mice were given 150 mg/kg *dl*‐PHPB by oral gavage for 2 months, which produced no effect on the level of serum glucose. In the Morris water maze test, KK‐A^y^ mice treated with *dl*‐PHPB showed significant improvements in spatial learning and memory deficits compared with vehicle‐treated KK‐A^y^ mice. Additionally, we performed 2D‐DIGE to compare brain proteomes of 5‐month KK‐A^y^ mice treated with and without *dl*‐PHPB. We found 14 altered proteins in the cortex and 11 in the hippocampus; two of the 25 altered proteins and another four proteins that were identified in a previous study on KK‐A^y^ mice were then validated by western blot to further confirm whether *dl*‐PHPB can reverse the expression levels of these proteins. The phosphoinositide 3‐kinase/protein kinase B/glycogen synthase kinase‐3β (PI3K/Akt/GSK‐3β) signaling pathway was also changed in KK‐A^y^ mice and *dl*‐PHPB treatment could reverse it.

**Conclusions:**

These results indicate that *dl*‐PHPB may play a potential role in diabetes‐associated cognitive impairment through PI3K/Akt/GSK‐3β signaling pathway and the differentially expressed proteins may become putative therapeutic targets.

## Graphical abstract

Among six confirmed proteins, HAGH, LAMTOR2, HINT1, DRP2, and EB1 are all related to PI3K/Akt/GSK‐3β signaling pathway. HAGH, LAMTOR2, HINT1, DRP2, and EB1 take part in PI3K/Akt/GSK3β signaling pathway. HINT can suppress the activity of GSK3β and play a role in neuroprotection. LAMTOR2 is crucial for Akt/mTOR signaling activation and influences endosomal biogenesis and receptor trafficking. HAGH takes part in scavenging system of methylglyoxal (MG) which is by PI3K/AKT/mTOR signaling. DRP2 is a brain‐specific substrate of GSK3 and Cyclin‐Dependent Kinase 5 (CDK 5) and takes part in axon elongation. GSK‐3β regulates the activity of CLIP‐associating protein 2 (CLASP2) and the interaction of CLASP2 and EB1 is necessary for the regulation of microtubule dynamics. G_o_ proteins are required for the activation of MAPKs and NF‐κB.

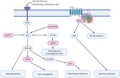

## 1 INTRODUCTION

Type 2 diabetes mellitus (T2DM) is a metabolic disorder and accounts for more than 90% of diabetes patients. As T2DM has an impact on cardiovascular, peripheral nervous systems, and central nervous system (CNS), and cognitive changes mainly affect learning and memory, mental speed and flexibility,^1^, ^2^ so diabetic encephalopathy (DE) is put forward. Because of the global prevalence of T2DM, DE has gained much public attention and has become a major public health concern worldwide. It is a series of neuropathological changes caused by diabetes and the common symptoms are paraesthesia, numbness, and impaired cognition.^3^

AD and T2DM are two age‐related diseases that are increasing worldwide. Alzheimer's disease (AD) is a most common neurodegenerative disorder. The accumulation of brain amyloid‐β (Aβ) and hyperphosphorylated tau is implicated in the pathogenesis of AD. Numerous studies have demonstrated that patients with diabetes have an increased risk of developing AD compared with healthy individuals.^1^, ^4^ However, the underlying biological mechanisms that link T2DM and AD are not fully understood. The aggregation of Aβ, disruption of the insulin signaling pathway, increased oxidative stress, dysregulated glucose metabolism, formation of advanced glycation end products (AGEs), and activation of inflammatory pathways all occur in both diseases.^5^, ^6^, ^7^, ^8^ Insulin resistance can affect energy metabolism, cell growth and differentiation, cellular repair mechanisms, and glucose utilization.^7^ It is a core feature of T2DM and seems to be the main common impairment of the two diseases.

Potassium 2‐(1‐hydroxypentyl)‐benzoate (*dl*‐PHPB) is the drug precursor of 3‐n‐butylphthalide (L‐NBP) that is synthesized by the Institute of Materia Medica, Chinese Academy of Medical Sciences. In 2009, *dl*‐PHPB was approved to undergo phase I, II, and III clinical trials by the State Food and Drug Administration as a new drug candidate for ischemic stroke.^9^, ^10^ Our preliminary studies showed that *dl*‐PHPB could improve cognitive deficits induced by hypoperfusion and also induced by icv infused with Aβ through preventing neuropathological alterations, inhibiting oxidative damage and inflammatory reactions.^10^ These results indicated that *dl*‐PHPB had the therapeutic potential for the treatment of vascular dementia (VaD) and AD.

Recently, a lot of evidence has shown that GSK‐3β may be the potential link between diabetes mellitus (DM) and AD.^11^ In DM, GSK‐3β is crucial for PI3K/Akt/insulin signaling pathway and is involved in the glucose metabolism in the brain.^12^ PI3K/Akt/GSK‐3β signaling pathway also plays an important role in hyperphosphorylation of the microtubule‐associated protein tau (tau) which is one of the important pathological features in AD.^13^ Our previous study showed that *dl*‐PHPB could upregulate PI3K/Akt signaling pathway and downregulated the expression of phosphorylated GSK‐3β in APP and presenilin 1 (PS1) double‐transgenic (APP/PS1) AD mice and further reduced hyperphosphorylation of tau protein.^14^

All existing evidence shows that *dl*‐PHPB has the potential to improve memory deficits in VaD and AD. Therefore, we speculate that it may also have a therapeutic effect on DE. The technique of 2D‐DIGE combined the traditional two‐dimensional electrophoresis technology with its characteristic of multiple fluorescence analysis technology. In this study, we investigated the pharmacological activity of *dl*‐PHPB on cognitive impairment in a T2DM animal model, KK‐A^y^ mice, and examined alterations in the cortical and hippocampal protein profile in mice with DE and in *dl*‐PHPB‐treated mice using 2D‐DIGE‐based proteomics. Moreover, we tested the PI3K/Akt/GSK‐3β pathway to investigate the possible underlying molecular mechanism.

## 2 MATERIALS AND METHODS

### 2.1 Animals and drug administration

**FIGURE 1:**
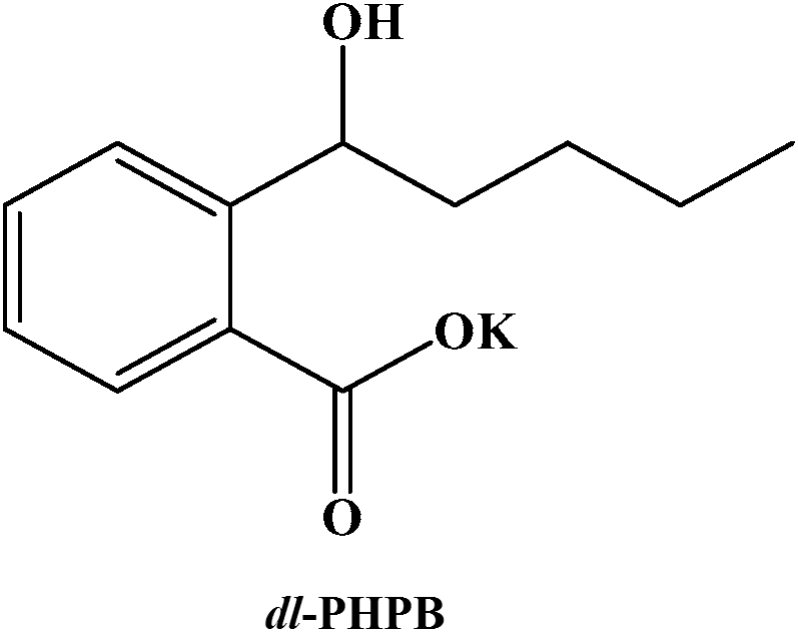
Chemical structure of *dl*‐PHPB

*dl*‐PHPB (purity > 98%) was offered by the Department of Medical Synthetic Chemistry, Institute of Material Medic (Beijing, China), and dissolved in distilled water. Its chemical structure is shown in Figure 1.

Adult male KK‐A^y^ mice and age‐matched C57 BL/6J mice were used in this study. Mice were randomly divided into three groups: C57 group (oral gavage distilled water), KK‐A^y^ group (oral gavage distilled water), and KK‐A^y^ + dl‐PHPB group (oral gavage *dl*‐PHPB 150 mg/kg). Distilled water and *dl*‐PHPB were administered to mice every day for a total of 2 months. Mice were kept in a temperature‐controlled room (23–25°C) under a 12‐h/12‐h light/dark cycle and free access to food and water. The C57 group was fed a standard diet and the other two groups were given a high‐fat diet (40% energy from fat) until the mice were sacrificed. Tail random blood glucose was measured when we obtained these mice and monitored every 2 weeks. All animal experiments were performed under the institutional guidelines and regulations of the Experimental Animal Center of the Chinese Academy of Medical Science, Beijing, China.

### 2.2 Morris water maze test

The Morris water maze test was proceeded to evaluate the mouse spatial learning and memory performance at the age of 21 weeks after *dl*‐PHPB administration. The device consists of a circular stainless steel water tank (120 cm in diameter and 50 cm in height). The tank was filled with water to a depth of 30 cm and the water's temperature was kept at 24 ± 1°C. A transparent acrylic platform (10 cm in diameter, hidden below the water surface about 1.5 cm) was placed at the center of the southwest quadrant during training. Obvious clues were on the walls of the room for the mice to orientate. The swimming paths were recorded by a video camera and data were automatically collected into a computer with an image analyzer immediately. The orientation navigation test lasted for 5 days with the location of the platform fixed, but the starting positions were changed every day. On each day, mice were trained with two consecutive trials and the intertrial interval was 30 s. The mice were permitted a maximum of 120 s to find the hidden platform and were allowed to rest on the platform for 30 s. If the mouse failed to climb on the platform itself within 120 s, it would be guided to the platform by the experimenter gently. During the test, the swimming pathway and escape latency of mice were recorded. On the 6th day, to assess long‐term memory consolidation, the platform was removed and the mice were placed in the pool from the opposite quadrant where the platform was located before. The mice were allowed to search for the platform for 120 s. The time that an animal crossed the position of the hidden platform at the first time, the time spent in the target quadrant and crossing times in the platform quadrant were recorded to measure the spatial memory maintenance.

### 2.3 Sample collection and preparation

After Morris water maze test, mice were anesthetized with 4% (w/w) chloral hydrate intraperitoneal injection (i.p.), and then were decapitated. Cortex and hippocampus were immediately dissected via surgery on ice and frozen in liquid nitrogen. All the samples were stored at −80°C until analysis.

Samples for 2D‐DIGE of each mouse were sonicated (5 × 10 s with an interval of 15 s) on ice in lysis buffer containing 30 mM Tris, 2 M thiourea, 7 M urea, 4% (w/v) CHAPS, 0.5% (v/v) IPG buffer (pH 3‐10NL; GE Healthcare, USA), and 1% protease inhibitor. The suspension was then centrifuged at 15,000 rpm for 30 min at 4 °C. The supernatant was collected and stored at −80°C until further use. Protein quantification was accurately conducted with a 2‐D Quant Kit (GE Healthcare).

Samples for western blot were sonicated (5 × 10 s with an interval of 15 s) on ice in lysis buffer containing 10 mM Tris, 1 mM EDTA, 1% (v/v) Triton, 0.1% (w/v) SDS, 0.2% (w/v) sodium deoxycholate, 101 mM Na_4_O_7_P_2_, 1 mM Na_3_VO_4_, and 1% protease inhibitor. The suspension was then centrifuged at 15,000 rpm for 30 min at 4°C. Protein quantification was performed with a BCA Quantification Kit (Applygen, China).

### 2.4 Proteins labeling with CyDyes and two‐dimensional electrophoresis

A quantity of 50 μg of protein sample from the cortex or hippocampus of each mouse (*n* = 6) was added to a tube and labeled with 400 pmol Cy3 or Cy5 minimal dye. To avoid possible dye bias, dyes were designed to swap within the two groups. A pooled internal standard consisting of equal amounts of each sample was created and labeled with Cy2 minimal dye. This internal standard would need to be sufficient to include on every gel. Centrifuged briefly in a microcentrifuge to make the solution collected at the bottom of the tube and left for 30 min on ice in the dark. Added 1 μl of 10 mM lysine to the sample and left for 10 min on ice in the dark to stop the reaction.

The main difference between 2D‐DIGE and standard 2‐DE (two‐dimensional electrophoresis) techniques is that up to three different protein samples can be run on a single gel. We mixed together three differently labeled protein samples (an internal standard labeled with Cy2, a diabetic sample and a control sample labeled with Cy3 or Cy5 respectively) and added an equal volume of 2× sample buffer containing 8 M urea, 2% (v/v) IPG buffer, 2% (w/v) DTT, 4% (w/v) CHAPS. The total volume of labeled protein was made up to 350 μl with the rehydration buffer consisting of 4% (w/v) CHAPS, 8 M urea, 1% (v/v) IPG buffer, 13mM DTT, and 0.002% (w/v) Bromophenol blue.

Isoelectric focusing was carried out using the Ettan IPGphor II IEF system (GE Healthcare) and 18 cm pH 3–10 nonlinear immobiline drystrips were used. IEF was run at 20°C overnight and finally achieving 60,000V h at a maximum current of 50 μA per strip. Second dimension separation was performed on an Ettan DALTsix Electrophoresis System (GE Healthcare) at 9°C using 2 W per gel for 1 h and then 15W per gel until the bromophenol blue dye front reached the bottom of the gel.

Gel image analysis was performed by using DeCyder 2D Software (GE Healthcare) which enables detection, quantification, matching and analysis of 2D‐DIGE system gels. The steps consist of the following processes: spot detection, background subtraction, in‐gel normalization, gel artifact removal, gel to gel matching, statistical analysis. Protein spots of interest with significant difference (average ratio ≥ 1.5 and *p* < 0.5) were filtered and a pick list was created. Then, we excised these proteins manually on a gel stained with Coomassie blue which separated unlabeled pooled protein samples for further mass spectrometry analysis.

### 2.5 LC‐MS/MS identification

Proteins were in‐gel digested with porcine trypsin. Then purified peptides were transferred to sample vials and analyzed using nano LC‐LTQ‐MS/MS (ThermoFinnigan, San Jose, CA) as previously described.^15^ BioWork (v. 3.3.1)/SEQUEST (v. 28) search engine and NCBI Mouse database were used to retrieve and identify proteins. Appraisal of each protein should have at least two specific peptides to support.

### 2.6 Western blot analysis

The expression levels of differentially expressed proteins were detected by western blot according to the protocol as described previously. 60 μg protein per lane was run on a polyacrylamide gel, transferred on to a PVDF membrane, blocked with 5% milk solution (nonfat dry milk in TBST) for 2 h and subsequently incubated with primary antibodies diluted in blocking solution overnight. The following primary antibodies were used in this study: rabbit monoclonal anti‐DRP2 (1:800; Abcam), rabbit monoclonal anti‐HAGH (1:1000; Cell Signal Technology), rabbit monoclonal anti‐Syn2 (1:800; Abcam), rabbit monoclonal anti‐LAMTOR2 (1:800; Abcam), mouse monoclonal anti‐EB1 (1:1000; Cell Signal Technology), rabbit monoclonal anti‐GNAO1 (1:800; Abcam), rabbit monoclonal anti‐HINT1 (1:800; Abcam), mouse monoclonal anti‐β‐actin (1:1000; Sigma Aldrich), rabbit monoclonal anti‐PI3K (1:1000; Cell Signaling), rabbit monoclonal Phospho‐Akt (Ser473) (1:1000; Cell Signaling), rabbit monoclonal Akt (1:1000; Cell Signaling), rabbit monoclonal Phospho‐GSK‐3β(Ser9) (1:1000; Cell Signaling), rabbit monoclonal GSK‐3β (1:1000; Cell Signaling).

### 2.7 Statistical analysis

Statistical tests were performed by SPSS version 16.0 software. All the data are expressed as mean ± *SEM*. Data normality was verified by Shapiro‐Wilk test. The results of behavioral tests were analyzed by two‐way repeated measures ANOVA followed by the Bonferroni post hoc test. One‐way ANOVA analysis followed by the Tukey post hoc test was performed to analyze statistical difference for multiple group comparisons. *p*‐Value < 0.05 was considered to be statistically significant.

## 3 RESULTS

### 3.1 Serum index analysis

**FIGURE 2:**
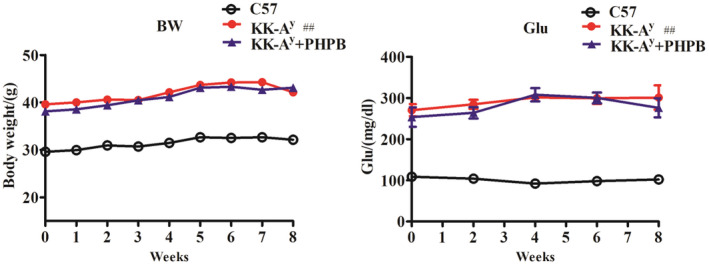
Body weight (BW), serum glucose (Glu) of C57 mice, KK‐A^y^ mice, and KK‐A^y^ + dl‐PHPB during drug application for 2 months. *n* = 15 for the C57 group, *n* = 20 for the KK‐A^y^ group, and *n* = 19 for KK‐A^y^ + dl‐PHPB group. ^##^ *p* < 0.01 vs C57 group

The body weight (BW) and serum glucose (Glu) levels were significantly higher (*p* < 0.01) in KK‐A^y^ mice than in C57 mice at 5 months of age (Figure 2). However, administration of *dl*‐PHPB to KK‐A^y^ mice had no effect on these indexes compared with KK‐A^y^ mice.

### 3.2 Cognitive performance in the Morris water maze test

**FIGURE 3:**
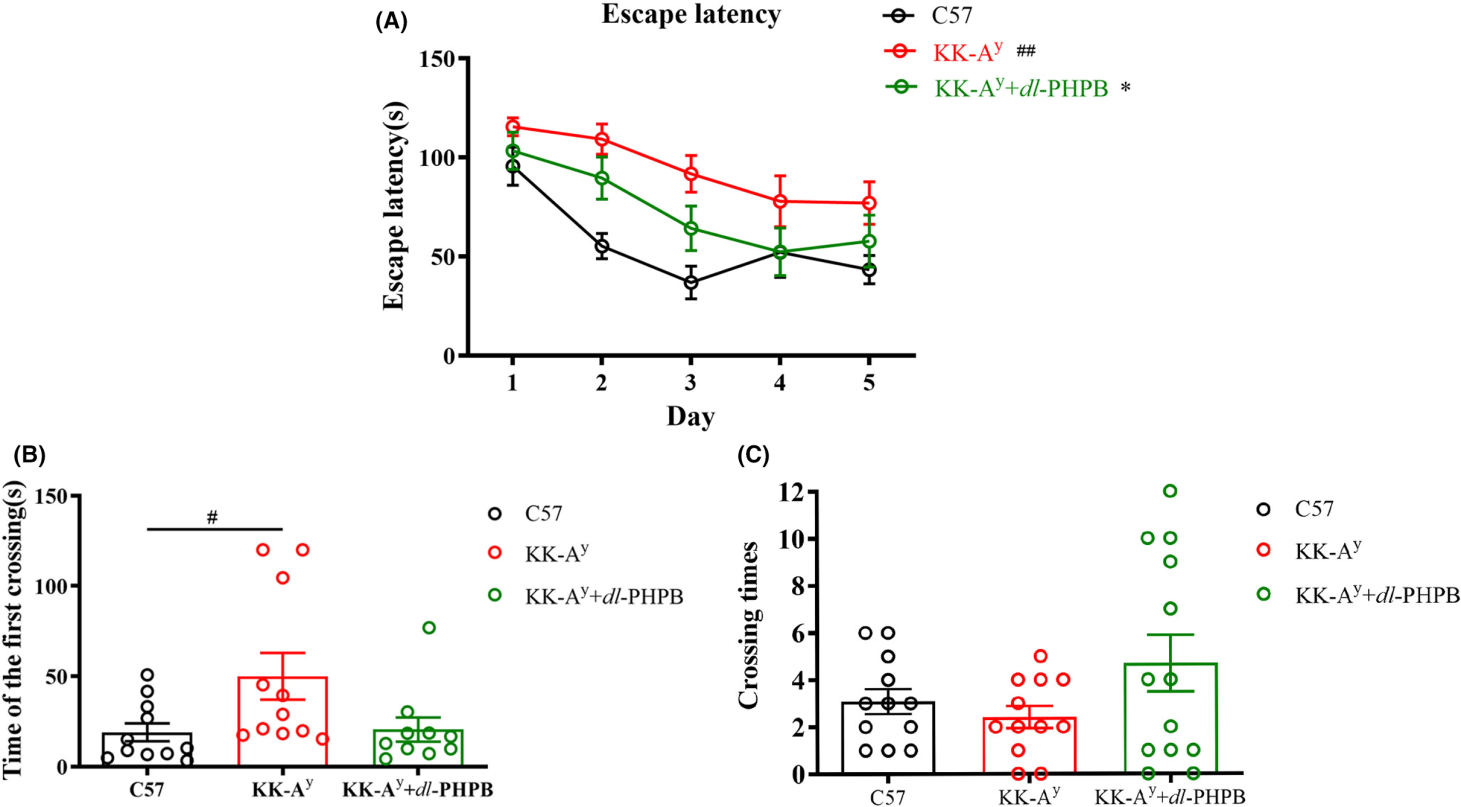
*dl*‐PHPB attenuates cognitive impairment of KK‐A^y^ mice in the Morris water maze test. (A) Time course of escape latency from day 1 to day 5. (B) The time that an animal crossed the position of the hidden platform at the first time on day 6. (C) Crossing times in the platform quadrant on day 6. *n* = 15 for C57 group and *n* = 20 for KK‐A^y^ group, *n* = 19 for KK‐A^y^ + dl‐PHPB group. ^##^ *p* < 0.01 and ^#^ *p* < 0.05 vs C57 group, ^*^ *p* < 0.05 vs KK‐A^y^ group

Our previous studies revealed that KK‐A^y^ mice showed cognitive deficits in the Morris water maze test and synaptic plasticity beginning at 3 months of age.^16^ To test the effect of *dl*‐PHPB on cognitive impairment, we evaluated the orientation navigation task using the Morris water maze. The spatial learning ability was measured by escape latency (the time required to reach the hidden platform). Figure 3A shows that during the 5 training days, the escape latency of the C57 group was shorter than that of the KK‐A^y^ group, and repeated‐measures ANOVA showed a significant effect of day on escape latency (*p* < 0.01), indicating spatial memory impairment. In contrast, KK‐A^y^ mice treated with *dl*‐PHPB showed reduced escape latency, indicating that *dl*‐PHPB can improve the spatial learning and memory impairment of KK‐A^y^ mice. In the probe trial, *dl*‐PHPB also increased the crossing times of platform location and decrease the first crossing time of platform location, as compared with KK‐A^y^ mice, but not significantly (Figure 3B,C). These results show that *dl*‐PHPB can attenuate the spatial learning and memory deficits of KK‐A^y^ mice.

### 3.3 Fluorescence‐based DIGE and protein identification in cortical tissue from KK‐A^y^ mice and KK‐A^y^ +dl‐PHPB mice

**FIGURE 4:**
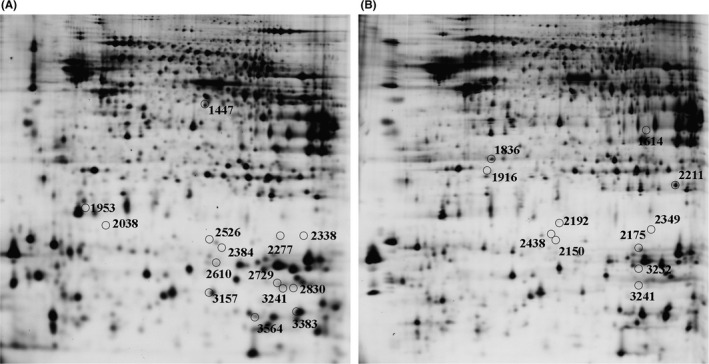
Distribution of altered proteins in the cortex (A) and hippocampus (B). Spots of differentially expressed proteins are labeled with black circles and corresponding numbers. *n* = 6 for each group

**FIGURE 5:**
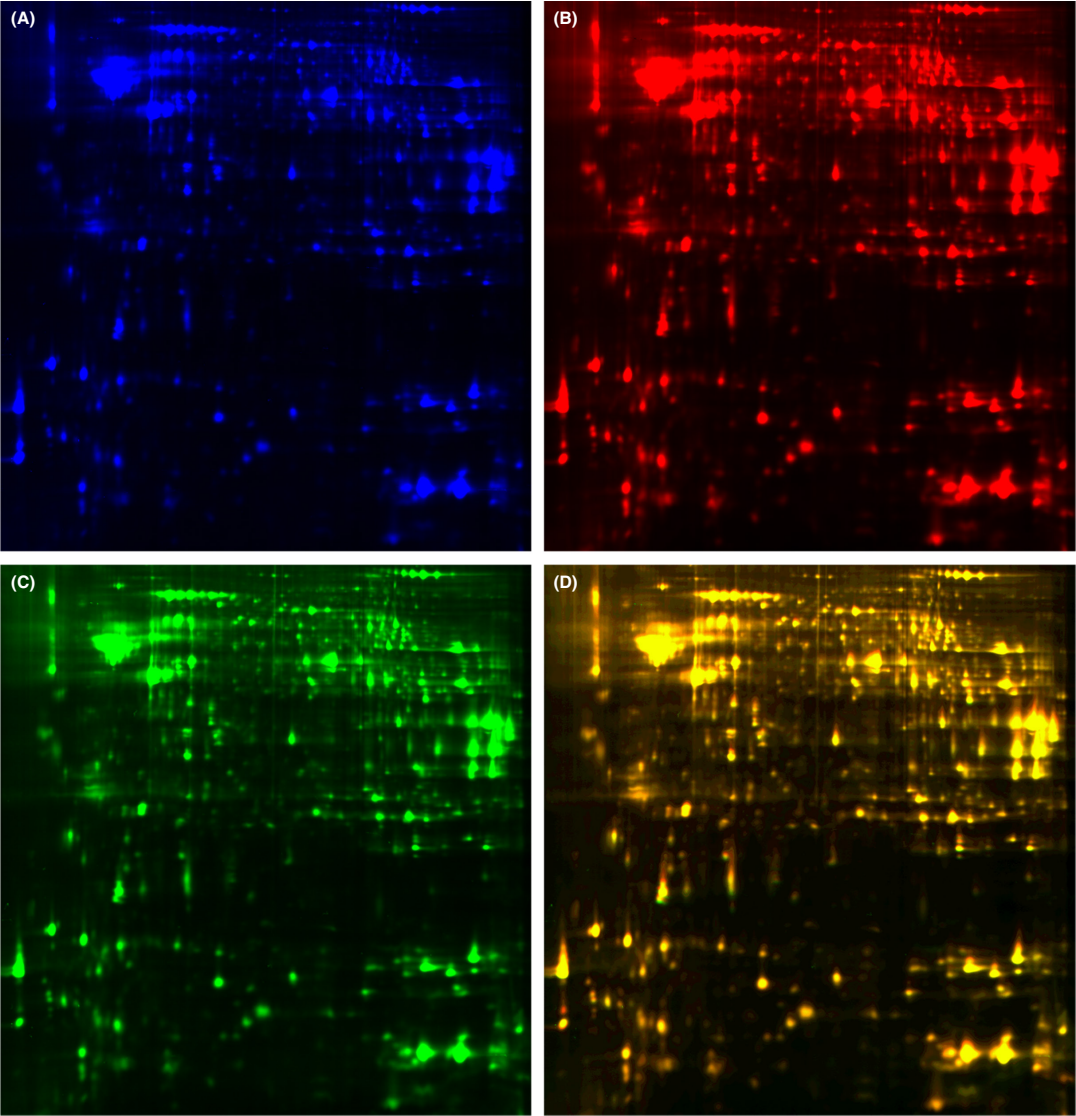
Representative image of 2D‐DIGE gels of cortex from 5‐month KK‐A^y^ mice and KK‐A^y^ + dl‐PHPB mice. (A) Cy2 (blue) image of proteins from an internal standard (equal amount of KK‐A^y^ group and KK‐A^y^ + dl‐PHPB group). (B) Cy5 (red) image of proteins from KK‐A^y^ group. (C) Cy3 (green) image of proteins from KK‐A^y^ + dl‐PHPB group. (D) The overlay image shows yellow spots indicating proteins with equal expression levels of the two groups, green spots and red spots indicating proteins with higher or downregulated expression

For each gel, more than 5000 spots were detected using DeCyder 2D software. Differentially expressed protein spots are labeled with black circles and corresponding numbers in Figure 4A. Figure 5 shows a representative 2D‐DIGE image of cortical tissue from 5‐month KK‐A^y^ mice and KK‐A^y^ + dl‐PHPB mice. The overlay image of Figure 5B and Figure 5C (Figure 5D) shows yellow spots, indicating proteins with equal expression levels in the two groups; green spots and red spots indicate proteins with up‐ or downregulated expression. A total of 14 altered proteins were identified by LC‐MS/MS, 12 (spot 1447, 1953, 2277, 2338, 2384, 2526, 2610, 2729, 2830, 3157, 3383, and 3564) of which were significantly upregulated (variation ratio ≥ +1.5, *p* < 0.05), and 2 (spot 3241, 2038) were significantly downregulated (variation ratio ≥ −1.5, *p* < 0.05) in the KK‐A^y^ + dl‐PHPB group compared with the KK‐A^y^ group. Detailed identification information of these proteins is shown in Table 1.

**TABLE 1:** Detailed identification information of differentially expressed proteins in cortex of KK‐A^y^ mice given *dl*‐PHPB compared with KK‐A^y^ mice

Spot NO.	Protein name	Accession GI No.	Sequence coverage (%)	Score	p*I*	MW (kD)	Average ratio	*p*‐ Value
1447	Guanine nucleotide‐binding protein G(o) subunit alpha	P18872	16.10	4.56	5.53	40.1	1.56	0.047
1953	Synaptosomal‐associated protein 25	P60879	55.83	36.03	4.77	23.3	1.82	0.044
2038	Chromobox protein homolog 1	P83917	22.16	9.82	4.93	21.4	−1.61	0.022
2277	U8 snoRNA‐decapping enzyme	Q6P3D0	35.38	16.94	7.12	21.8	2.16	0.043
2338	Protein lin‐7 homolog C	O88952	22.34	6.29	8.43	21.8	3.01	0.0067
2384	Guanosine‐3’,5'‐bis(diphosphate) 3'‐pyrophosphohydrolase MESH1	Q9D114	29.05	11.54	5.96	20.2	2.20	0.00074
2526	Rab‐like protein 5	Q9DAI2	23.78	4.27	5.25	20.8	2.01	0.047
2610	Diphosphoinositol polyphosphate phosphohydrolase 3‐alpha	P0C027	32.93	3.59	5.69	18.6	1.69	0.0035
2729	Actin‐related protein 2/3 complex subunit 5‐like protein	A3KGQ6	24.18	6.27	6.80	17.1	3.07	0.038
2830	Vacuolar protein sorting‐associated protein 29	D3Z645	36.81	1.73	7.01	16.1	2.01	0.027
3157	14 kDa phosphohistidine phosphatase	Q9DAK9	32.26	4.44	5.53	14.0	1.87	0.0022
3241	Cytochrome c oxidase subunit 5A	P12787	21.92	3.65	6.54	16.1	−1.58	0.042
3383	Peptidyl‐prolyl cis‐trans isomerase FKBP1A	P26883	29.63	4.55	8.16	11.9	1.81	0.039
3564	Hemoglobin subunit beta‐1 (Fragment)	E9Q223	12.62	2.84	6.37	11.1	2.46	0.013

Note

Variation ratio, the ratio of spot intensity of the KK‐A^y^ mice + dl‐PHPB group to that of the same spot in the KK‐A^y^ group; “ +”and “ –” represent “upregulation” and “downregulation.”

Abbreviations: MW, molecular mass in kDa; p*I*, isoelectric point.

### 3.4 Fluorescence‐based DIGE and protein identification in hippocampal tissue from KK‐A^y^ mice and KK‐A^y^ +dl‐PHPB mice

**FIGURE 6:**
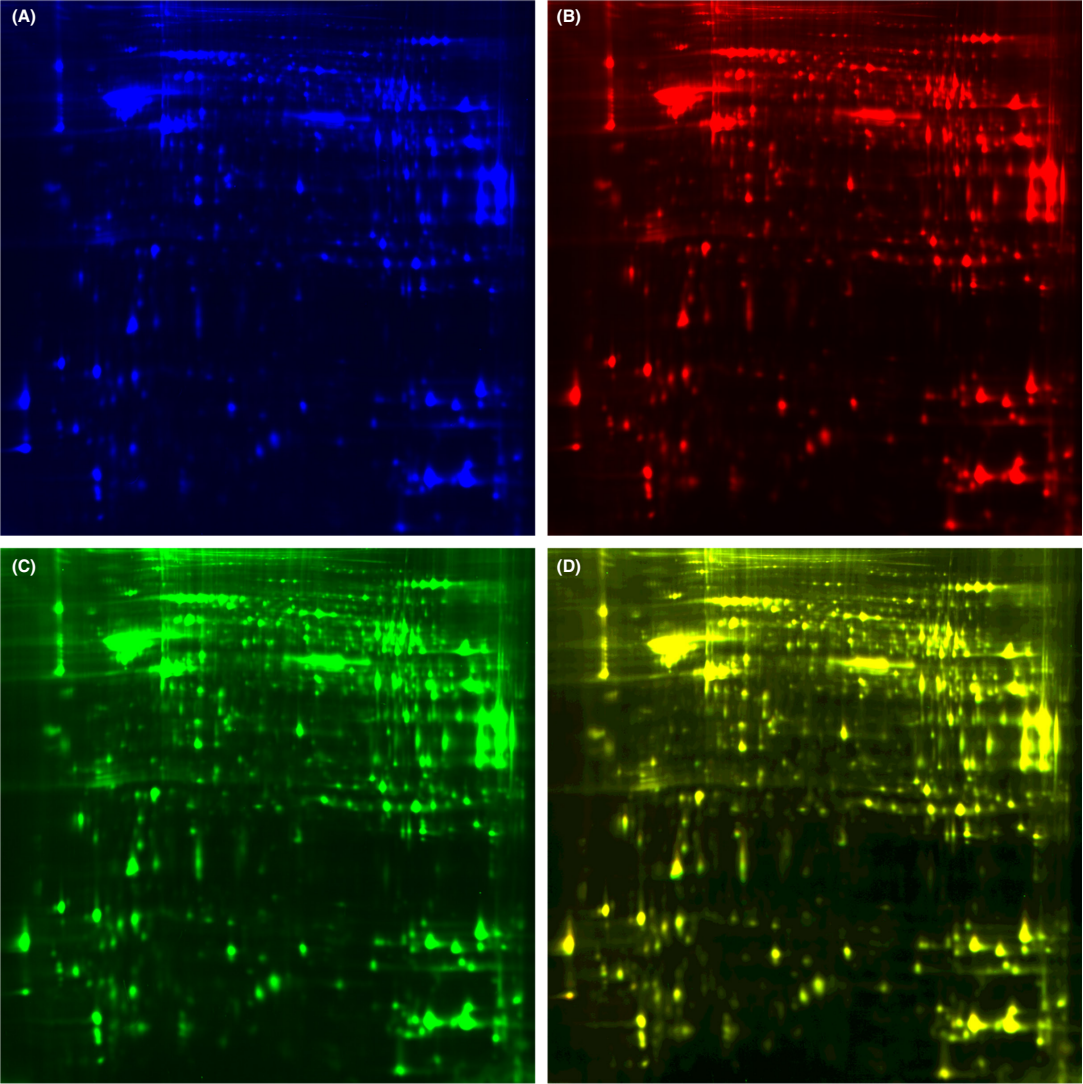
Representative image of 2D‐DIGE gels of hippocampus from 5‐month KK‐A^y^ mice and KK‐A^y^ + dl‐PHPB mice. (A) Cy2 (blue) image of proteins from an internal standard (equal amount of KK‐A^y^ group and KK‐A^y^ + dl‐PHPB group). (B) Cy5 (red) image of proteins from KK‐Ay group. (C) Cy3 (green) image of proteins from KK‐A^y^ + dl‐PHPB group. (D) The overlay image shows yellow spots indicating proteins with equal expression levels of the two groups, green spots and red spots indicating proteins with higher or downregulated expression

Figure 6 shows a representative 2D‐DIGE image of hippocampal tissue from the two groups. Differentially expressed protein spots are labeled with black circles and corresponding numbers in Figure 4B. A total of 11 altered proteins were identified by LC‐MS/MS, 10 (spot 1614, 1836, 1916, 2150, 2175, 2192, 2211, 2349, 2438, and 3241) of which were significantly downregulated (variation ratio ≥ +1.5, *p* < 0.05), and 1 (spot 3232) was significantly upregulated (variation ratio ≥ −1.5, *p* < 0.05) in the KK‐A^y^ + dl‐PHPB group compared with the KK‐A^y^ group. Detailed identification information of these proteins is shown in Table 2.

**TABLE 2:** Detailed identification information of differentially expressed proteins in hippocampus of KK‐A^y^ mice given *dl*‐PHPB compared with KK‐A^y^ mice

Spot NO.	Protein name	Accession GI No.	Sequence coverage (%)	Score	p*I*	MW	Average ratio	*p*‐ Value
1614	Elongation factor Ts	Q9CZR8	9.57	7.24	7.06	35.3	−2.15	0.048
1836	Triosephosphate isomerase	P10649	32.11	12.50	5.74	32.2	−1.53	0.032
1916	Apolipoprotein A‐I	Q00623	25.76	18.35	5.73	30.6	−1.56	0.045
2150	Lactoylglutathione lyase	Q9CPU0	51.09	25.00	5.47	20.8	−1.51	0.011
2175	Protein deglycase DJ‐1	A2A813	18.29	3.67	6.54	18.5	−1.62	0.033
2192	Ras‐related protein Rab‐6B	P61294	10.10	5.46	5.53	23.4	−2.24	0.0078
2211	Cytochrome b‐c1 complex subunit Rieske	Q9CR68	8.03	2.28	8.70	29.3	−2.09	0.0044
2349	Transgelin	Q9R1Q8	46.73	22.49	7.33	22.5	−3.25	0.030
2438	Peroxiredoxin‐2	Q61171	65.15	51.52	5.41	21.8	−2.53	0.0010
3232	Histidine triad nucleotide‐binding protein 1	P70349	18.25	2.48	6.87	13.8	2.04	0.0030
3241	Fatty acid‐binding protein	Q05816	50.37	4.42	6.54	15.1	−3.41	0.042

Note

Variation ratio, the ratio of spot intensity of the KK‐A^y^ mice + dl‐PHPB group to that of the same spot in the KK‐A^y^ group; “+” and “–” represent “upregulation” and “downregulation.”

Abbreviations: MW, molecular mass in kDa; p*I*, isoelectric point.

### 3.5 Confirmation of differentially expressed proteins by western blot

**FIGURE 7:**
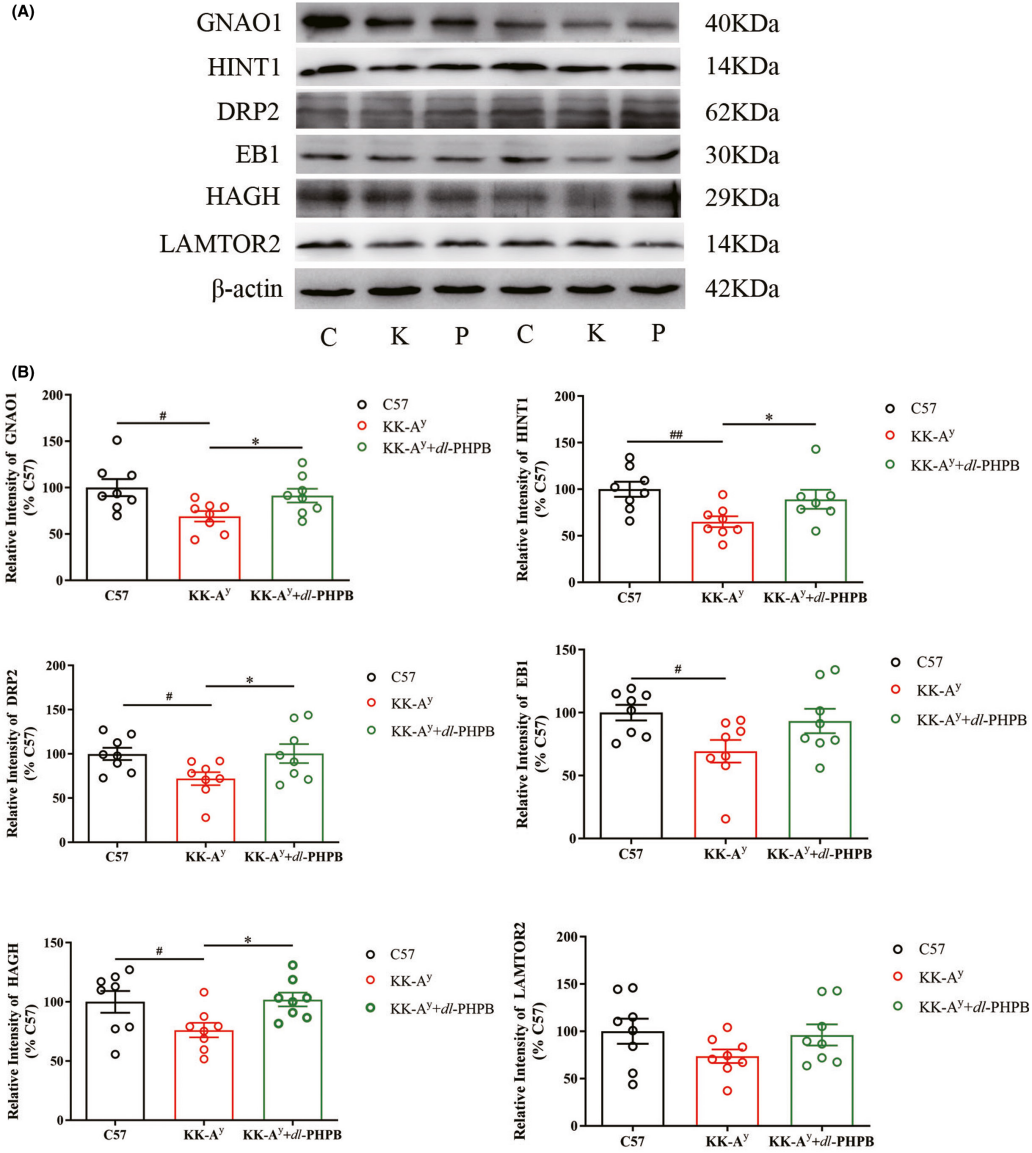
Validation of differentially expressed proteins in the cortex of C57 mice, KK‐A^y^ mice and KK‐A^y^ mice + dl‐PHPB via western blot. (A) Representative western blot images of Gnao1, HINT1, DRP2, EB1, HAGH, and LAMTOR2 in the cortex. (B) Quantitative analysis of Gnao1, HINT1, DRP2, EB1, HAGH, and LAMTOR2. Quantified results were normalized to β‐actin expression. Values were expressed as percentages compared with the control group (set to 100%). Data are represented as mean ± *SEM* and *n* = 7–8 mice per group. ^#^ *p* < 0.05 vs C57 group, ^*^ *p* < 0.05 vs KK‐A^y^ group

A total of 25 proteins were altered in the cortex and hippocampus, and two (Guanine nucleotide‐binding protein G(o) subunit alpha (GNAO1); Histidine triad nucleotide‐binding protein 1 (HINT1)) were chosen for validation via western blot. We also examined another four proteins (hydroxyacylglutathione hydrolase (HAGH); microtubule‐associated protein RP/EB family member 1(EB1); dihydropyrimidinase‐related protein 2 (DRP2); Mitogen‐activated protein‐binding protein‐interacting protein (LAMTOR2)) that were confirmed to be altered in KK‐A^y^ mice in our previous study to observe their variations after *dl*‐PHPB treatment. Representative immunoblot images (Figure 7A) and bar graphs (Figure 7B) of the brain cortex homogenates of C57 mice, KK‐A^y^ mice, and KK‐A^y^ +dl‐PHPB mice are displayed. GNAO1, HINT1, HAGH, EB1, and DRP2 were significantly downregulated in the cortex of KK‐A^y^ mice compared with C57 mice, and LAMTOR2 expression was also downregulated but without significance. After *dl*‐PHPB treatment, all six proteins tended to be upregulated compared with the KK‐A^y^ group, and GNAO1, HINT1, DRP2, and HAGH were significantly upregulated.

**FIGURE 8:**
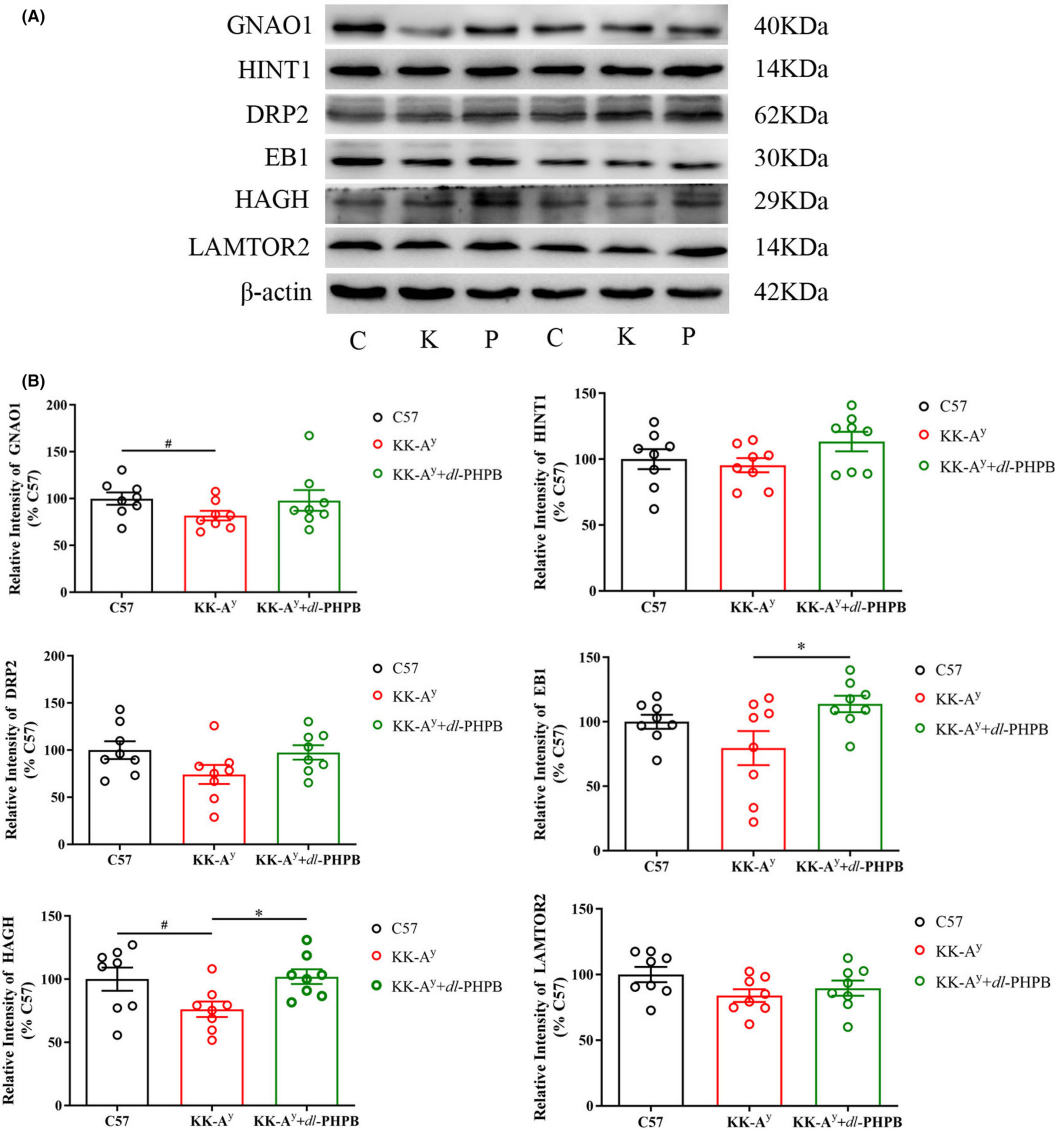
Validation of differentially expressed proteins in the hippocampus of C57 mice, KK‐A^y^ mice and KK‐A^y^ mice + dl‐PHPB via western blot. (A) Representative western blot images of Gnao1, HINT1, DRP2, EB1, HAGH, and LAMTOR2 in the hippocampus. (B) Quantitative analysis of Gnao1, HINT1, DRP2, EB1, HAGH, and LAMTOR2. Quantified results were normalized to β‐actin expression. Values were expressed as percentages compared with the control group (set to 100%). Date were represented as mean ± *SEM* and *n* = 7–8 mice per group. ^#^ *p* < 0.05 vs C57 group, ^*^ *p* < 0.05 vs KK‐A^y^ group

We also examined the expression levels of the six proteins (GNAO1, HINT1, HAGH, EB1, DRP2, and LAMTOR2) in the hippocampus. Representative immunoblot images (Figure 8A) and bar graphs (Figure 8B) of the hippocampal brain homogenates of C57 mice, KK‐A^y^ mice, and KK‐A^y^ +dl‐PHPB mice are displayed. Compared with the C57 group, the expression of the six proteins in the KK‐A^y^ group tended to be downregulated, but only GNAO1 and HAGH were significantly downregulated. In the KK‐A^y^ + dl‐PHPB group, only the expression levels of EB1 and HAGH were significantly upregulated compared with the KK‐A^y^ group (Figure 8B).

### 3.6 PI3K/Akt/GSK‐3β signaling pathway in the brain of KK‐A^y^ mice and KK‐A^y^ +dl‐PHPB mice

**FIGURE 9:**
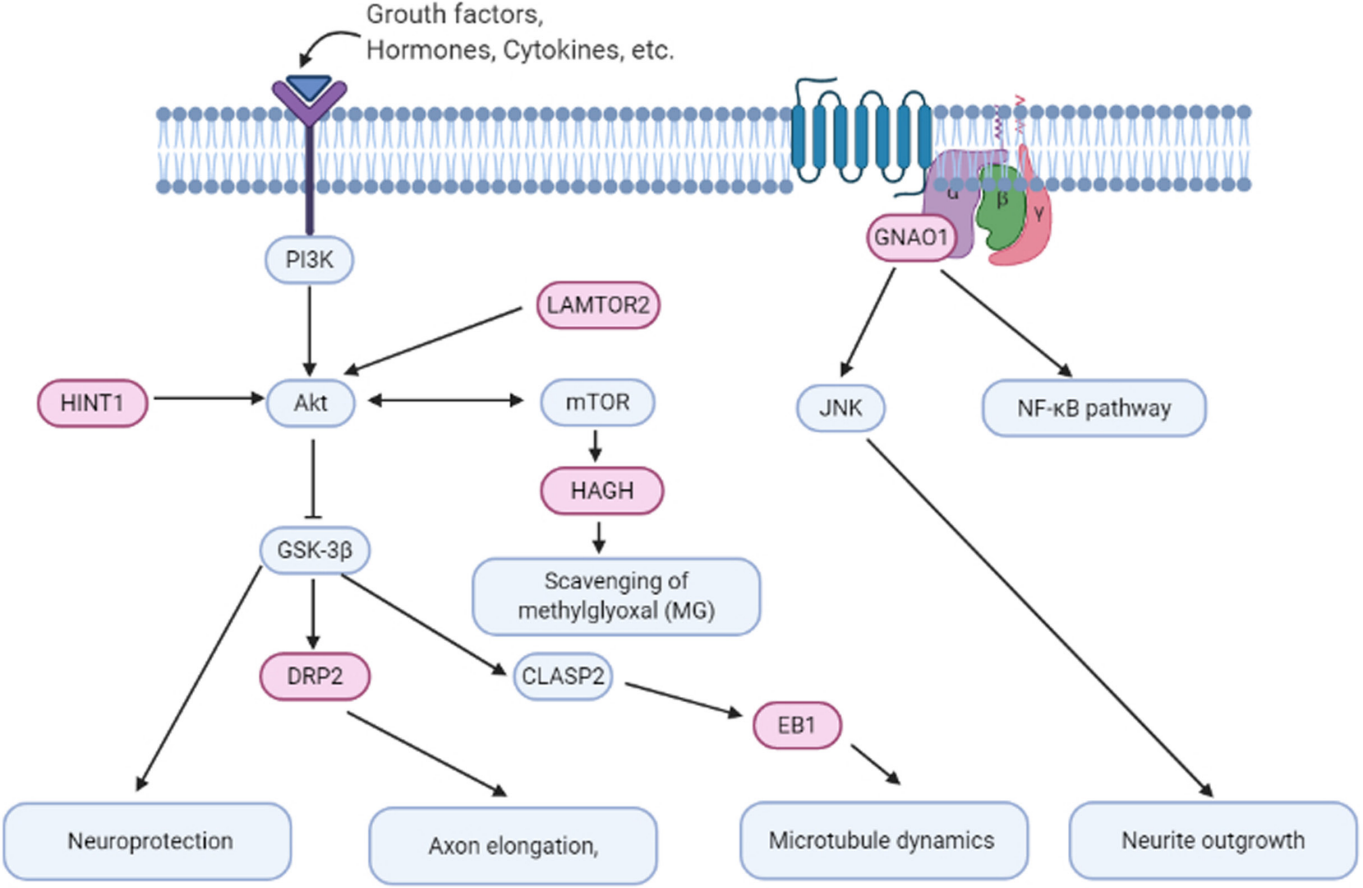
HINT1, HAGH, EB1, DRP2, and LAMTOR2 take part in PI3K/Akt/ GSK3β signaling pathway. HINT can suppress the activity of GSK3β and play a role in neuroprotection. LAMTOR2 is crucial for Akt/mTOR signaling activation and influences endosomal biogenesis and receptor trafficking. HAGH takes part in the scavenging system of methylglyoxal (MG) which is by PI3K/AKT/mTOR signaling. DRP2 is a brain‐specific substrate of GSK3 and Cyclin‐Dependent Kinase 5 (CDK 5) and takes part in axon elongation. GSK‐3β regulates the activity of CLIP‐associating protein 2 (CLASP2) and the interaction of CLASP2 and EB1 is necessary for the regulation of microtubule dynamics. G_o_ proteins are required for the activation of MAPKs and NF‐κB

**FIGURE 10:**
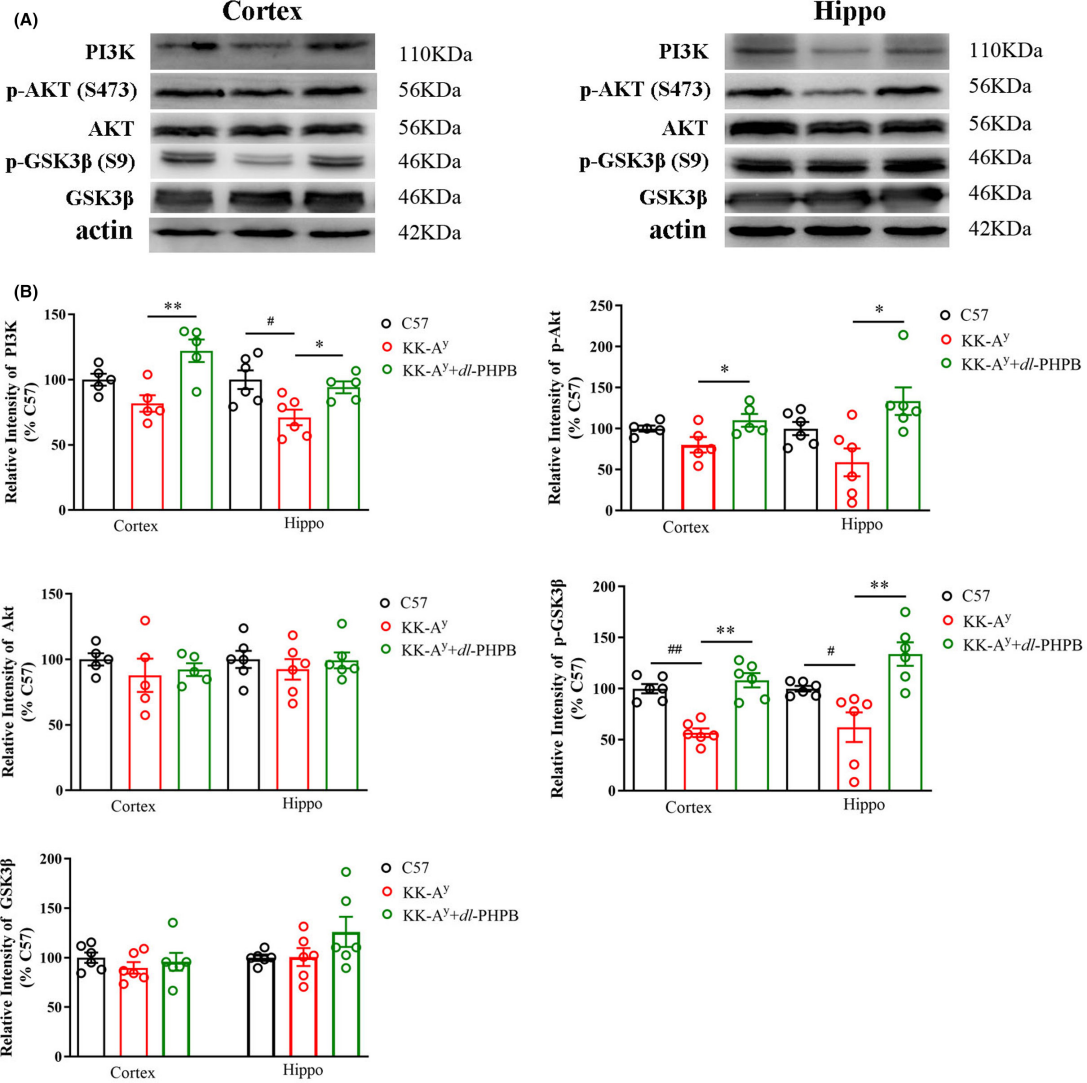
Validation of PI3K/Akt/GSK‐3β signaling pathway in the cortex and hippocampus of C57 mice, KK‐A^y^ mice and KK‐A^y^ mice + dl‐PHPB via western blot. (A) Representative western blot images of PI3K, p‐Akt (S473), Akt, GSK3β(S9), GSK3β in the cortex and hippocampus. (B) Quantitative analysis of PI3K, p‐Akt (S473), Akt, GSK3β(S9), and GSK3β. Quantified results were normalized to β‐actin expression. Values were expressed as percentages compared with the C57 group (set to 100%). Data are represented as mean ± *SEM* and *n* = 5–6 mice per group. ^#^ *p* < 0.05 vs C57 group, ^##^ *p* < 0.01 vs C57 group, ^*^ *p* < 0.05, ^**^ *p* < 0.01 vs KK‐A^y^ group

Studies have shown that the PI3K/Akt/GSK‐3β signaling pathway is important in neuroprotection and can enhance cell survival by inhibiting apoptosis and stimulating cell proliferation.^17^, ^18^ And GSK‐3β takes an important part in the pathology both of DM and AD. Our previous studies also showed that L‐NBP improved cognitive impairment in an APP/PS1transgenic AD mouse model might through inhibitory effects of CDK‐5 and GSK‐3β signaling pathways.^19^ Furthermore, as shown in Figure 5, 9, 5 (HAGH, LAMTOR2, HINT1, DRP2, EB1) of the 6 confirmed proteins are all related to PI3K/Akt/GSK‐3β signaling pathway.^20^, ^21^, ^22^, ^23^, ^24^, ^25^ Based on this, we explored the PI3K/Akt/GSK‐3β signaling pathway in the cortex and hippocampus of KK‐A^y^ mice and KK‐A^y^ + dl‐PHPB mice by western blot assay. As shown in Figure 10, PI3K, p‐AKT (Ser473), p‐GSK3β (Ser9) were all downregulated both in the cortex and hippocampus of KK‐A^y^ mice compared with C57 mice, but only p‐GSK3β (Ser9) was significant. After *dl*‐PHPB treatment, all the three proteins were significantly upregulated compared with the KK‐A^y^ group. However, there were no differences between the expression of total Akt and GSK3β, and *dl*‐PHPB also had no effect on the expression levels of the two proteins.

## 4 DISCUSSION

*dl*‐PHPB is thought to be a promising candidate for the treatment of stroke and AD, and it has been demonstrated to improve memory deficits in several animal models. However, the effect of *dl*‐PHPB on DE has not been examined. Our present study used proteomic technology to compare differentially expressed proteins in brain tissues of KK‐A^y^ mice with and without *dl*‐PHPB treatment to identify the potential targets of *dl*‐PHPB in DE treatment. *Dl*‐PHPB treatment by oral gavage for 2 months could significantly reverse the memory deficits of KK‐A^y^ mice exhibited in the Morris water maze test, but it had no effect on the level of serum glucose. DIGE analysis identified 14 spots in the cortex and 11 in the hippocampus that were different between the KK‐A^y^ and KK‐A^y^ + dl‐PHPB groups, and western blot assays also confirmed these changes. And the effect of *dl*‐PHPB reversing memory deficits might be through improving PI3K/Akt/GSK‐3β signaling pathway.

Several studies have demonstrated that the induction of diabetes in mouse models leads to an acceleration of neuropathology and cognitive dysfunction.^26^, ^27^, ^28^ DE is a series of behavioral and pathological changes caused by constitutive hyperglycemia, including cognitive decline, neuronal loss, and disorders of glycolipid metabolism.^29^, ^30^ KK‐A^y^ mice, as a major model of T2DM and obesity with a polygenic background, are regarded as suitable models for exploring the mechanisms of T2DM with obesity and its complications.^31^, ^32^ Compared with control C57 mice, KK‐A^y^ mice had higher body weight, serum glucose levels, and *dl*‐PHPB treatment had no effect on these indexes. In behavioral tests, KK‐A^y^ mice showed significantly impaired learning and memory ability; however, *dl*‐PHPB could reverse cognition impairment in KK‐A^y^ mice. These results indicated that *dl*‐PHPB‐induced improvements in the spatial learning and memory impairment of KK‐A^y^ mice might not involve regulation of the serum glucose level.

We used 2D‐DIGE to conduct a proteomic study of brain tissues of KK‐A^y^ mice treated with and without *dl*‐PHPB to identify proteins that might participate in the development of DE, as well as identify putative targets of *dl*‐PHPB in the treatment of DE. More than 5000 protein spots were successfully detected in brain tissues of the two groups on each gel, and a total of 25 proteins showed significant changes (13 upregulated and 12 downregulated). These proteins were then categorized into five groups according to their functions based on the UniProt database (Table 3). Most proteins were involved in energy metabolism, neuronal structure and trafficking, protein metabolism, stress and repair, and cell signaling.

**TABLE 3:** Function of differentially expressed proteins in the cortex and hippocampus of the two groups

Function	Identified protein	Changed in		Up/downregulated after *dl*‐PHPB administration
		cortex	hippo	
Energy metabolism	Guanosine‐3’,5'‐bis(diphosphate) 3'‐pyrophosphohydrolase MESH1	✓		↑
	Rab‐like protein 5	✓		↑
	Elongation factor Ts		✓	↓
	Triosephosphate isomerase		✓	↓
	Apolipoprotein A‐I		✓	↓
	Cytochrome c oxidase subunit 5A	✓		↓
	Fatty acid‐binding protein		✓	↓
Neuronal structure and trafficking	Synaptosomal‐associated protein 25	✓		
	Chromobox protein homolog 1	✓		↓
	U8 snoRNA‐decapping enzyme	✓		↑
	Actin‐related protein 2/3 complex subunit 5‐like protein	✓		↑
	Myelin basic protein (Fragment)	✓		↑
	Transgelin		✓	↓
	Ras‐related protein Rab‐6B		✓	↓
	Cytochrome b‐c1 complex subunit Rieske		✓	↓
Stress and repair	Lactoylglutathione lyase		✓	↓
	Peroxiredoxin‐2		✓	↓
Protein metabolism	Vacuolar protein sorting‐associated protein 29	✓		↑
	14 kDa phosphohistidine phosphatase	✓		↑
	Histidine triad nucleotide‐binding protein 1		✓	↑
	Peptidyl‐prolyl cis‐trans isomerase FKBP1A	✓		↑
	Protein deglycase DJ‐1		✓	
Cell signaling	Guanine nucleotide‐binding protein G(o) subunit alpha	✓		↑
	Protein lin‐7 homolog C	✓		↑
	Diphosphoinositol polyphosphate phosphohydrolase 3‐alpha	✓		↑

Note

The classification of protein function was based on Uni‐Prot database (http://www.uniprot.org/).

Two of the differentially expressed proteins (GNAO1 and HINT1) were chosen to be validated via western blot. Furthermore, another four proteins (HAGH, EB1, DRP2, and LAMTOR2) that we confirmed to be altered in KK‐A^y^ mice in our previous study were also examined to observe their variation after *dl*‐PHPB treatment, even though no changes were detected in the proteomic experiment. All six proteins were downregulated in brain tissues in the KK‐A^y^ group compared with control group mice, and *dl*‐PHPB treatment could partially reverse the decrease in these six proteins.

Guanine nucleotide‐binding protein G(o) subunit alpha (GNAO1), is a component of G protein transmembrane signaling.^33^ It is highly enriched in the CNS and can inhibit neurotransmitter release and exhibit a protective effect on the CNS. Mutations in GNAO1 were previously associated with neurologic pathophysiology.^34^ A previous proteomic analysis of AD also showed that GNAO1 was altered in the human hippocampus and inferior parietal lobe.^33^, ^35^ Furthermore, GNAO1 was also found to be downregulated in the retina of diabetic mice.^36^ GNAO1 gene's mutations can cause a complex constellation of neurological disorders including epilepsy, movement disorders, and developmental delay.^37^ In our study, proteomic and western blot analyses both showed that GNAO1 was downregulated in the brains of KK‐Ay mice, and *dl*‐PHPB could reverse this decrease in GNAO1. This result indicated that *dl*‐PHPB might improve the spatial memory of KK‐A^y^ mice through regulation of transmembrane signaling and cellular processes.

Histidine triad nucleotide‐binding protein 1 (HINT1) belongs to the histidine triad superfamily, which contains a highly conserved His‐X‐His‐X‐His‐XX motif (X indicates a hydrophobic amino acid).^38^ HINT1 is widely expressed in different tissues, such as the liver, kidney, and brain.^39^ The function of this protein is not fully known, but it is reported to be an inhibitor of Ca^2+^‐dependent protein kinase C (PKC) and may play a role in tumor suppression.^40^, ^41^ The downregulation of HINT1 has also been reported in diabetes, AD, and schizophrenia.^42^, ^43^, ^44^ All these results suggest that HINT1 may be of importance to neuronal function, the exact physiological and cellular function of HINT1 in central nervous system remains to be deciphered.

Hydroxyacylglutathione hydrolase (HAGH) belongs to the glyoxalase system and helps prevent the formation of AGEs.^45^ This protein was reported to be altered in diabetic patients, animal models of diabetes,^45^, ^46^ and in AD patients.^47^ DRP2, also known as collapsin response mediator protein 2 (CRMP2), is a neuron‐specific phosphoprotein that plays roles in axon growth and elongation, establishing and maintaining neuronal polarity, and promoting microtubule assembly.^48^, ^49^ DRP2 was reported to be altered in AD patients, animal models of AD, and diabetic animal models.^50^, ^51^, ^52^ Microtubule‐associated protein RP/EB family member 1 (MAPRE1), also known as end‐binding protein 1 (EB1), belongs to the MAPRE gene family, which regulates microtubule functions and dynamics.^53^ Overexpression of EB1 could facilitate axonogenesis and complement the neurogenesis function of microtubule‐associated protein 1B (MAP1B).^54^ Ragulator complex protein LAMTOR2 (LAMTOR2) plays a role in regulating endosomal rearrangement, growth factor signaling, and proliferation, and its reduction leads to a decrease in the function of melanocytes, cytotoxic T cells, B cells, and neutrophils.^55^, ^56^ Neither EB1 nor LAMTOR2 were previously reported to be involved in AD and T2DM. In the present study, *dl*‐PHPB could upregulate the expression levels of these four proteins in KK‐A^y^ mice, indicating that *dl*‐PHPB might participate in preventing the formation of AGEs, axon growth and genesis, and regulation of microtubule functions and dynamics.

Resent, PI3K/Akt signal transduction system is paid attention in cancer and in neurodegenerative diseases. AKT is a downstream serine–threonine protein kinase of PI3K. PI3K is activated by signals from tyrosine kinases and G protein‐coupled receptors (GPCRs) and then promotes phosphoinositide‐dependent protein kinase 1 (PDK1) phosphorylated Akt protein on the Thr308 and Ser473, thereby activating Akt.^11^ Activated Akt then induces the downstream molecule GSK‐3β to transpose into cell membrane, and phosphorylates its N‐terminal Ser9 active site to inactivate GSK‐3β.^57^ Dysfunction of PI3K/Akt/GSK‐3β signaling pathway will not only lead to insulin deficiency and insulin resistance in DM patients, but also lead to tau hyperphosphorylation in the brain of AD patents.^58^, ^59^ Activation of PI3K/Akt pathway was effective against AD‐like lesions^60^ and could also prevent DM‐related pathological changes in DM mice.^61^ These findings are consistent with our experimental results. In KK‐A^y^ mice, the expressions of phosphor‐Akt and PI3K were obviously lower than those in control C57 mice, but phosphorylated GSK‐3β at Ser9 site was significantly higher. In contrast, *dl*‐PHPB significantly activated the PI3K‐Akt pathway by upregulating the phosphorylation levels of PI3K and Akt and downregulating the Ser9 phosphorylation level of GSK‐3β.

This study showed for the first time that *dl*‐PHPB administration could significantly improve spatial learning and memory deficits in a diabetic animal model. In addition, we employed DIGE to analyze the cortical and hippocampal proteome in KK‐A^y^ mice treated with and without *dl*‐PHPB and identified proteins that might be the effective molecular targets of *dl*‐PHPB. Western blot analysis further confirmed the results of the proteomic analysis and demonstrated that above protein expressions were regulated by *dl*‐PHPB. Especially, PI3K‐Akt signaling pathway could be an important target of *dl*‐PHPB treatment.

In summary, we performed a DIGE‐based proteomic analysis and successfully identified novel candidate proteins that might be involved in the development of DE. Potential intervention targets and molecular mechanisms of *dl*‐PHPB in brain tissues from KK‐A^y^ mice were also defined. Furthermore, we are the first to find a medicine that can upregulate the expression levels of HINT1, EB1, and LAMTOR2. Further studies are required to investigate the exact role of these proteins and their potential as therapeutic targets of DE.

## CONFLICT OF INTEREST

The authors declare no conflict of interest.

## Supporting information

Supplementary Material

## Data Availability

The data that support the findings of this study are available from the corresponding author upon reasonable request.
