# Supplementary material for: The attenuation effect of potassium 2‐(1‐hydroxypentyl)‐benzoate in a mouse model of diabetes‐associated cognitive decline: The protein expression in the brain
Source: CNS Neurosci Ther. 2022 Apr 20;28(7):1108–23. doi: 10.1111/cns.13847 (PMC9160457; doi:10.1111/cns.13847)

1. Full unedited gel/blot for Figure 7

a. GNAO1

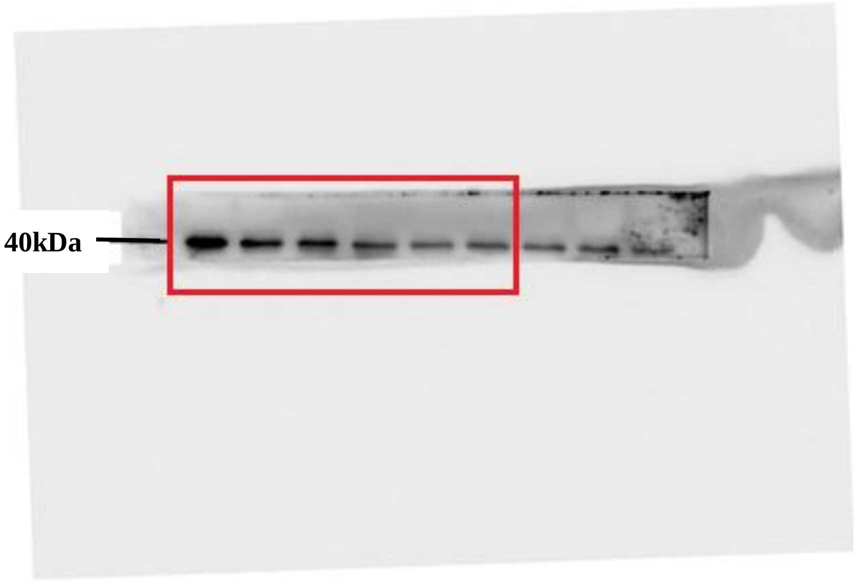

b. HINT11

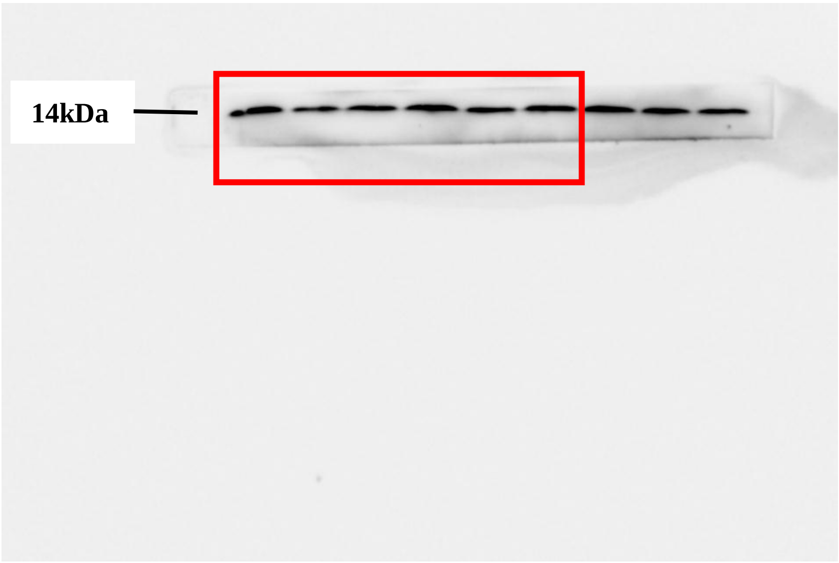

c. DRP2

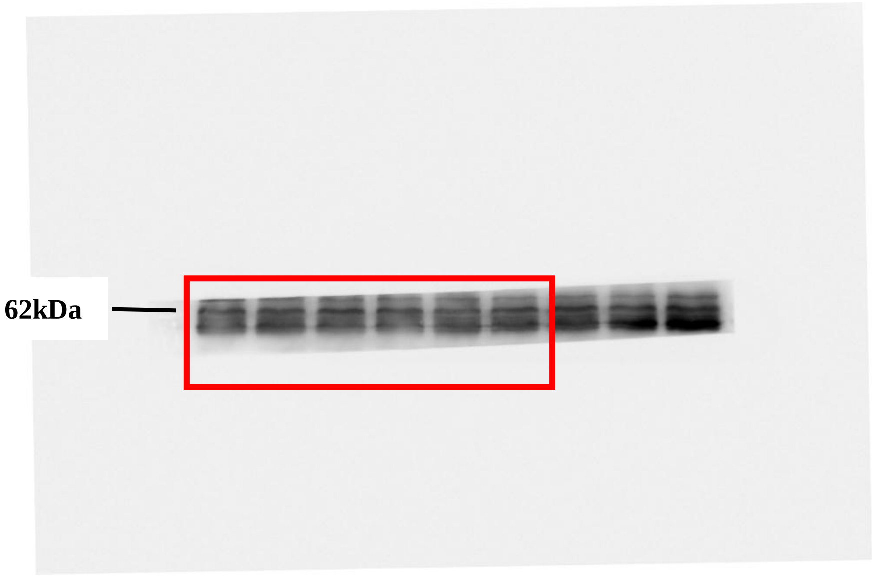

d. EB1

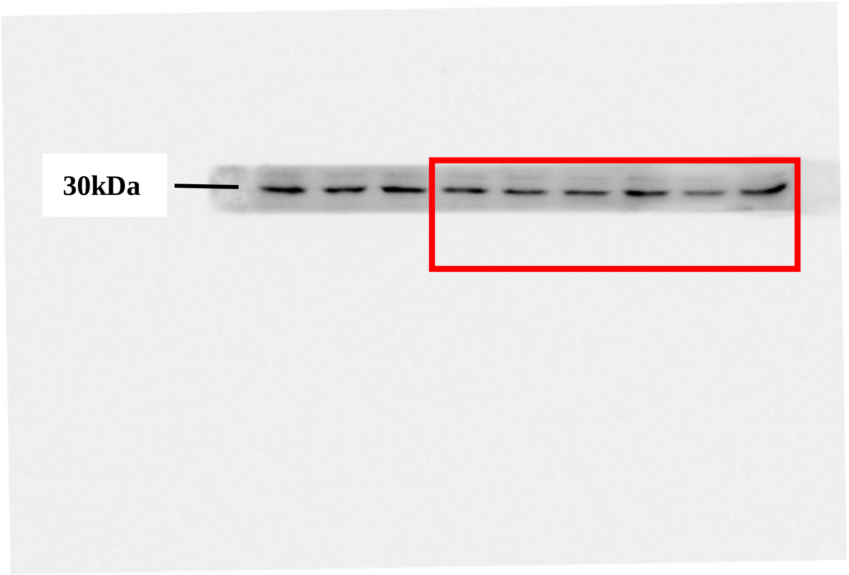

e. HAGH

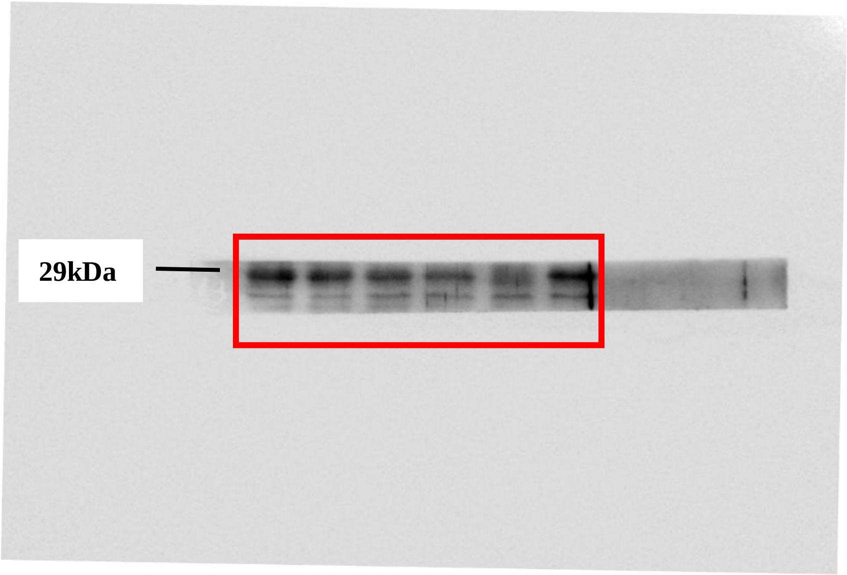

f. LAMTOR2

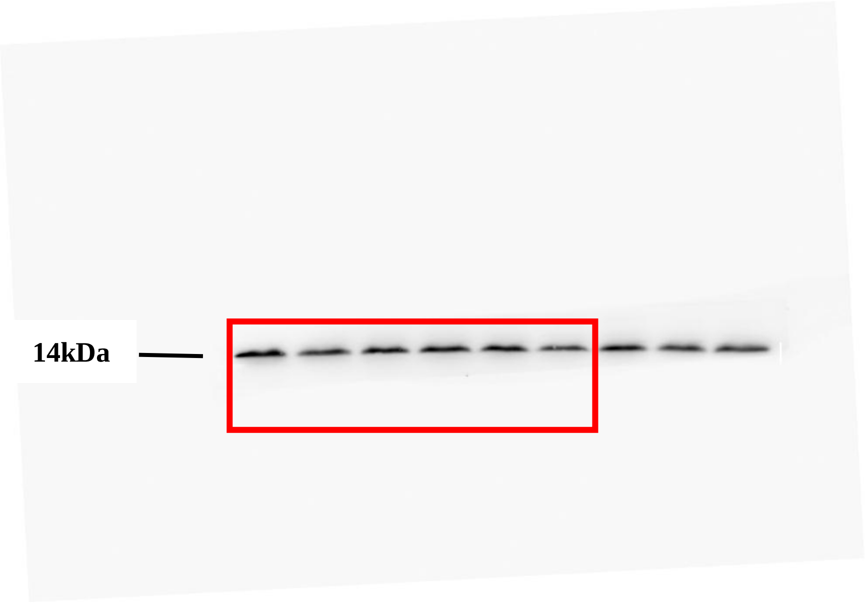

g. actin

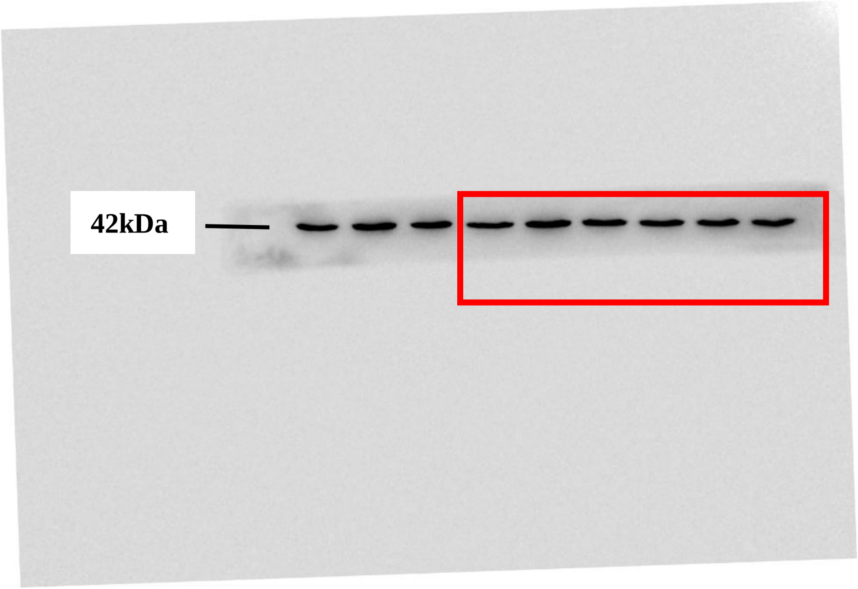

2. Full unedited gel/blot for Figure 8

a. GNAO1

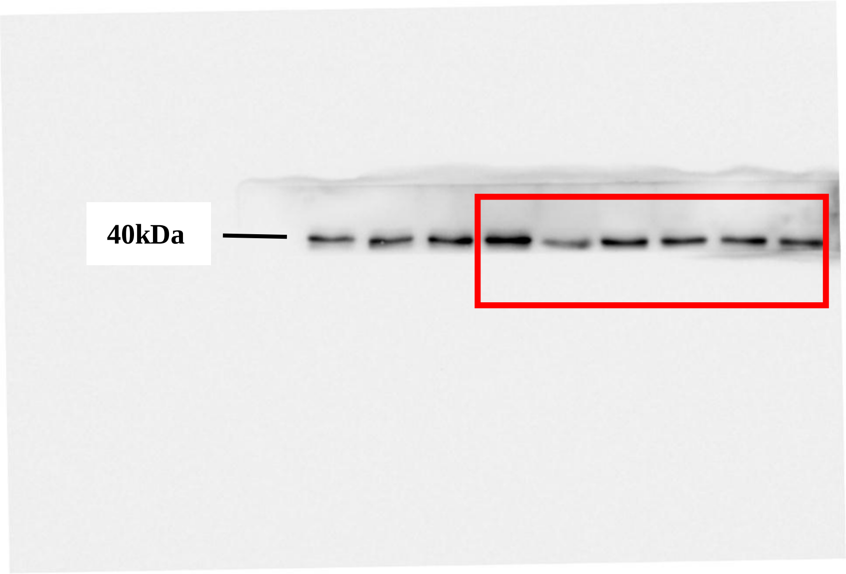

b. HINT11

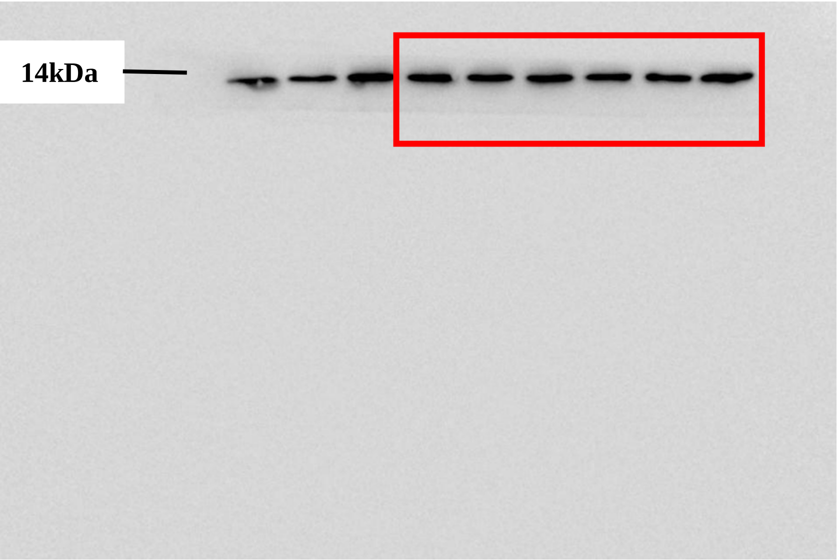

c. DRP2

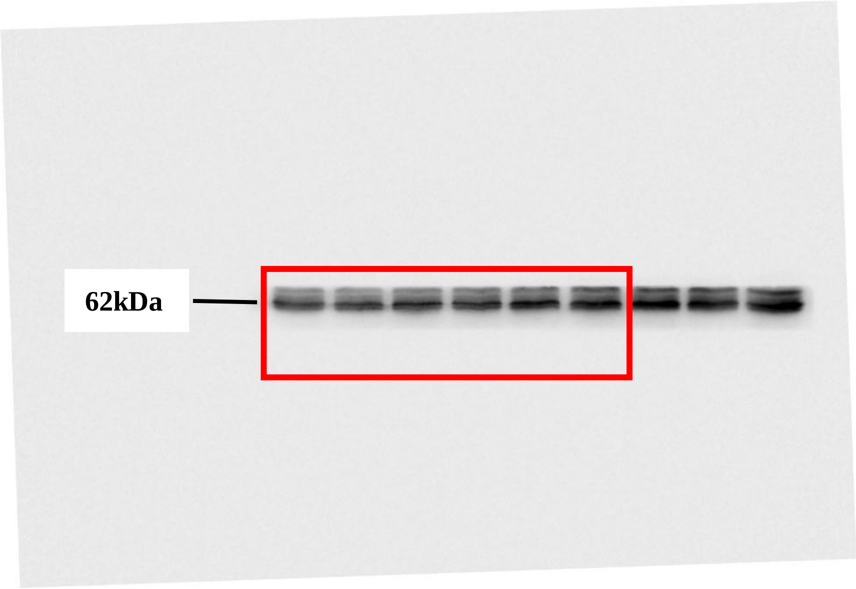

d. EB1

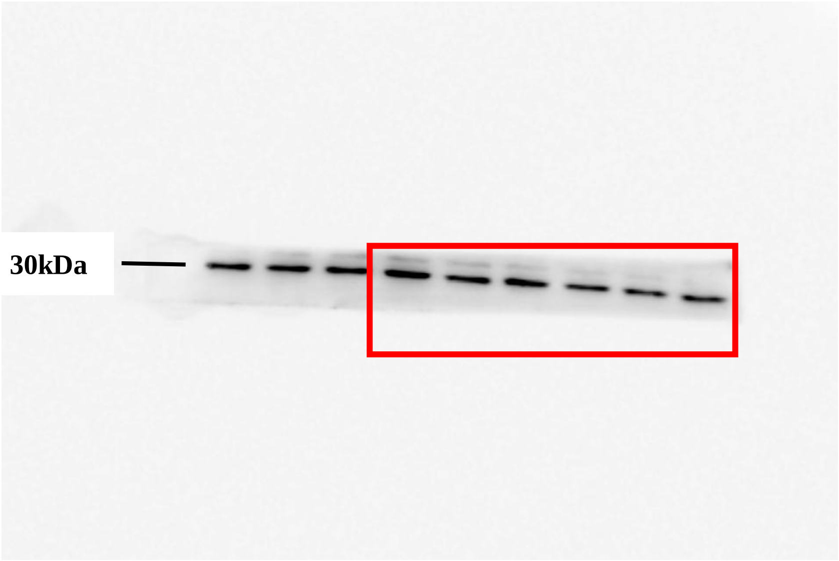

e. HAGH

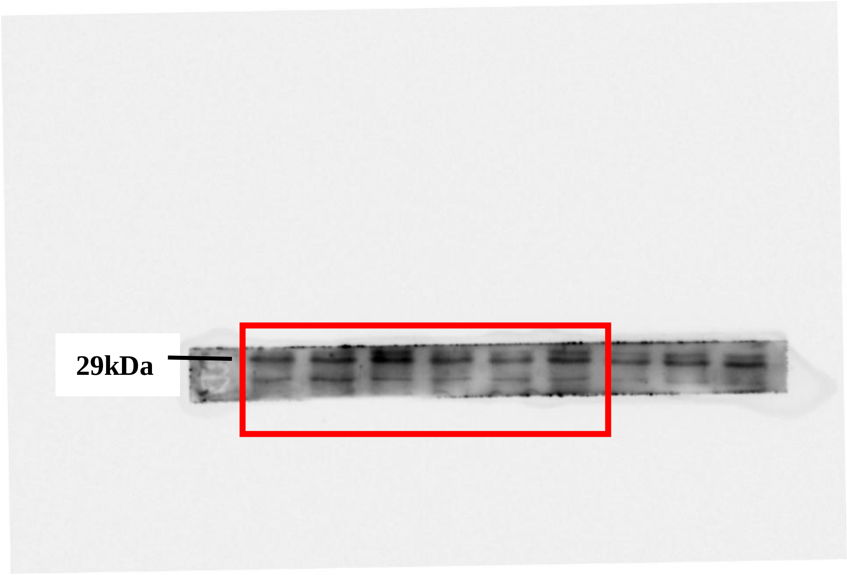

f. LAMTOR2

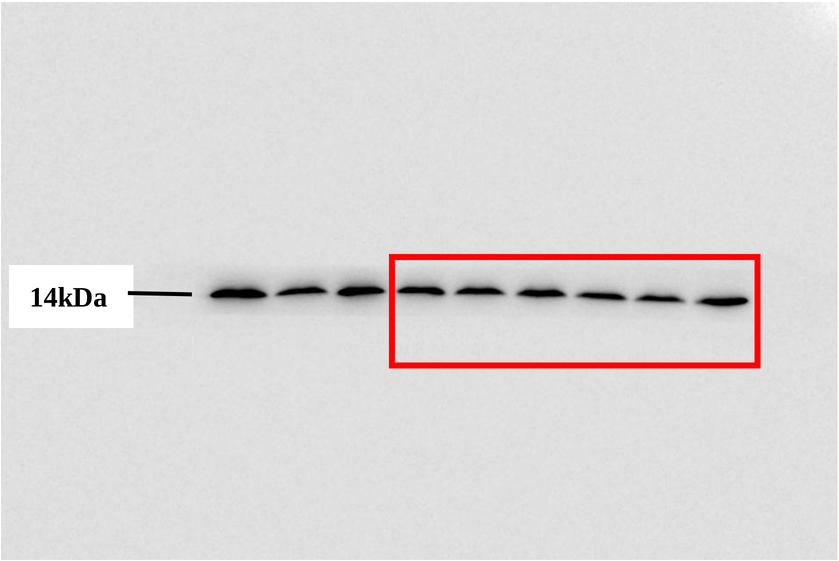

g. actin

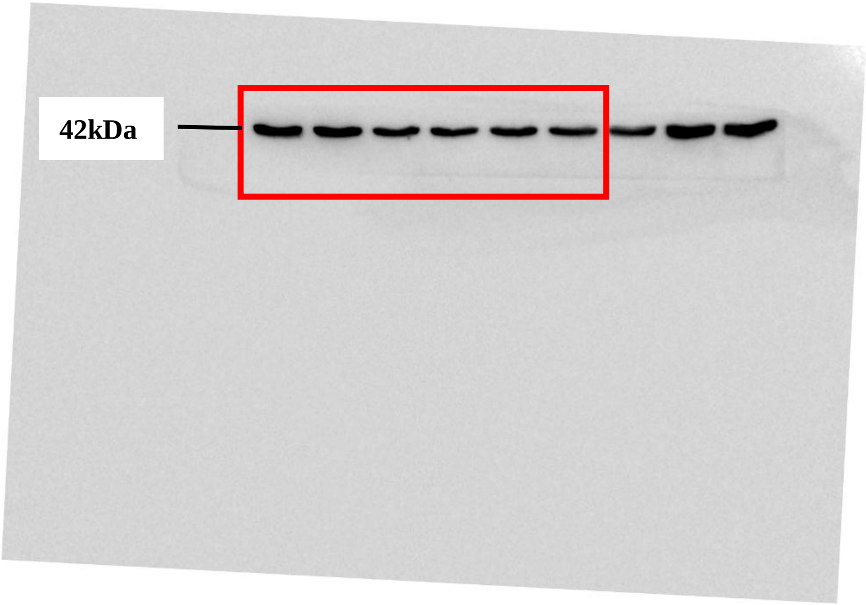

3. Full unedited gel/blot for Figure 10

a. PI3K-Cortex

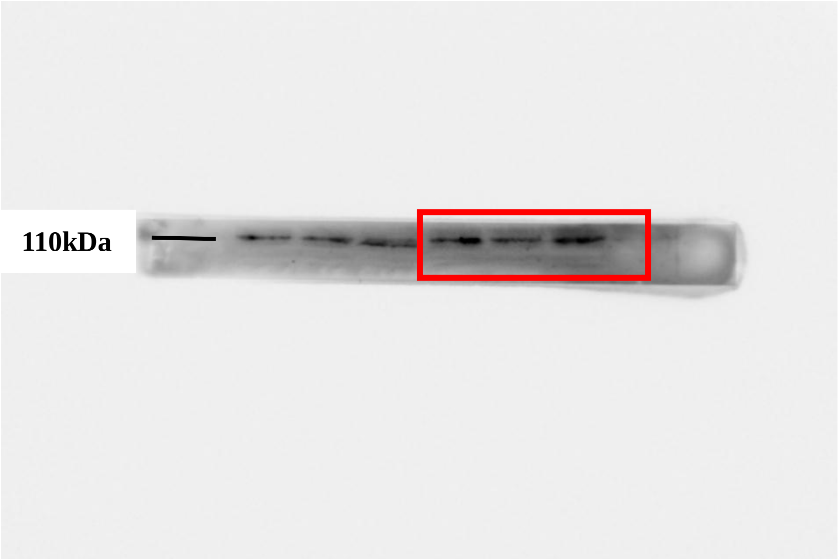

b. p-Akt-Cortex

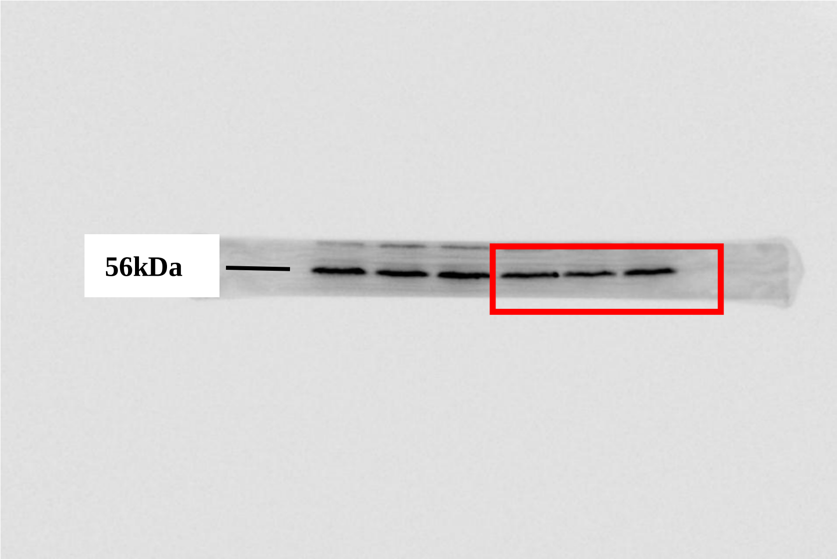

c. Akt-Cortex

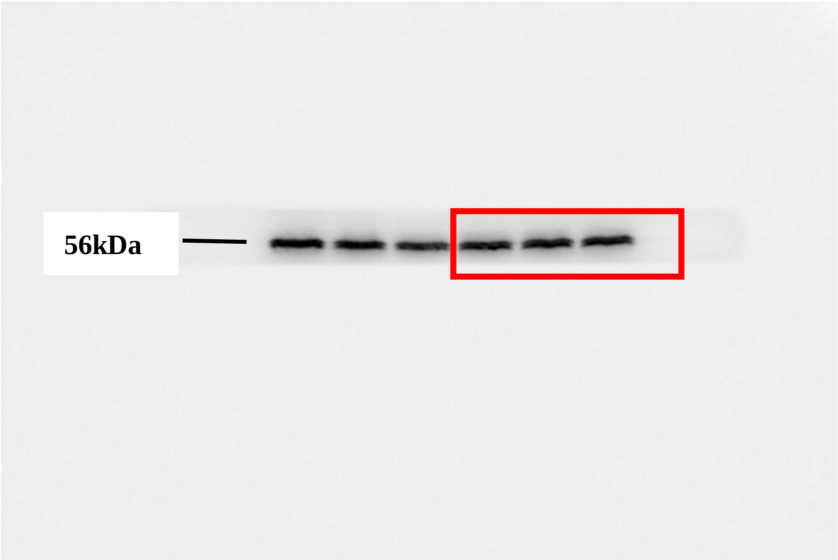

d. p-GSK3β-Cortex

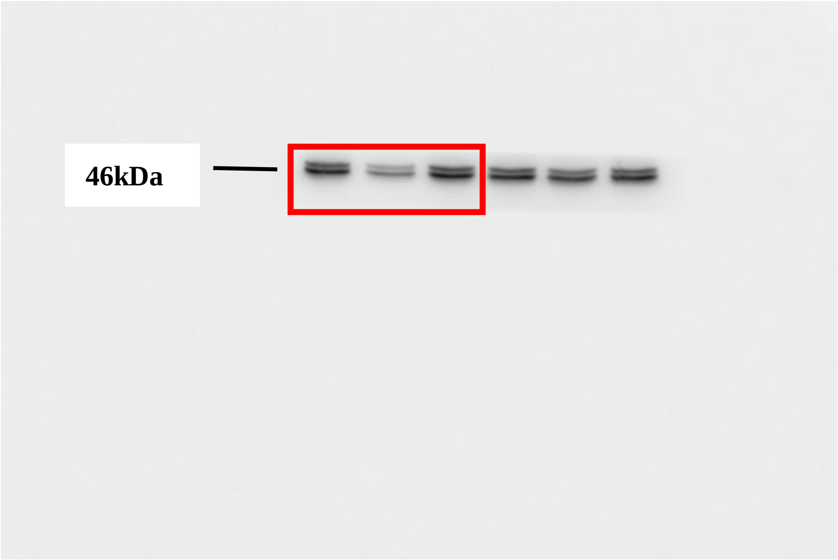

e. GSK3β-Cortex

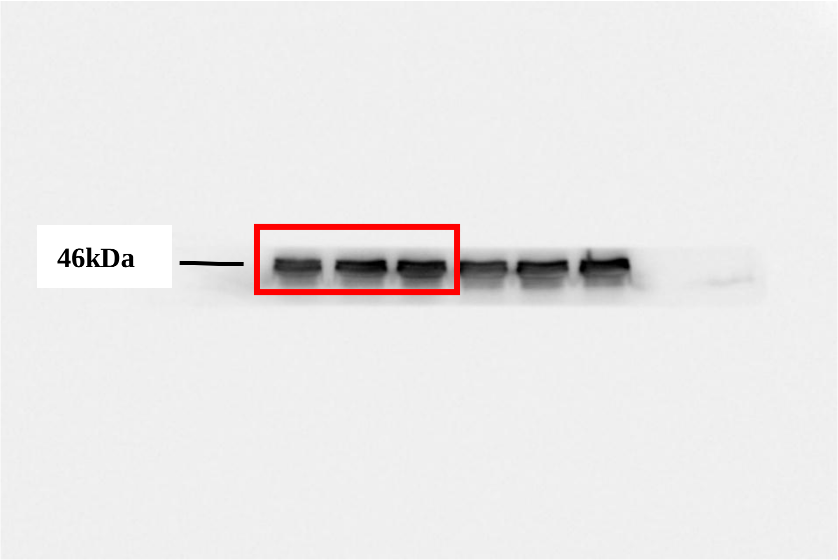

f. actin-Cortex

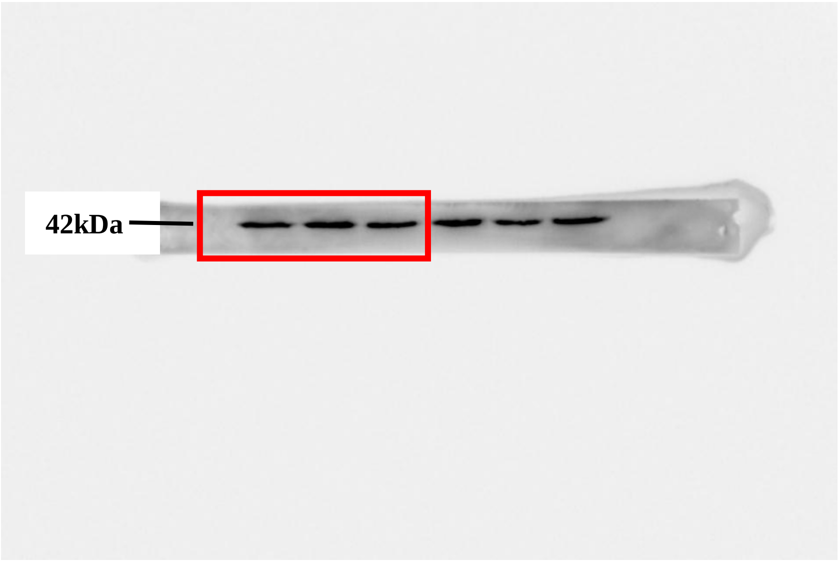

g. PI3K -Hippocampus

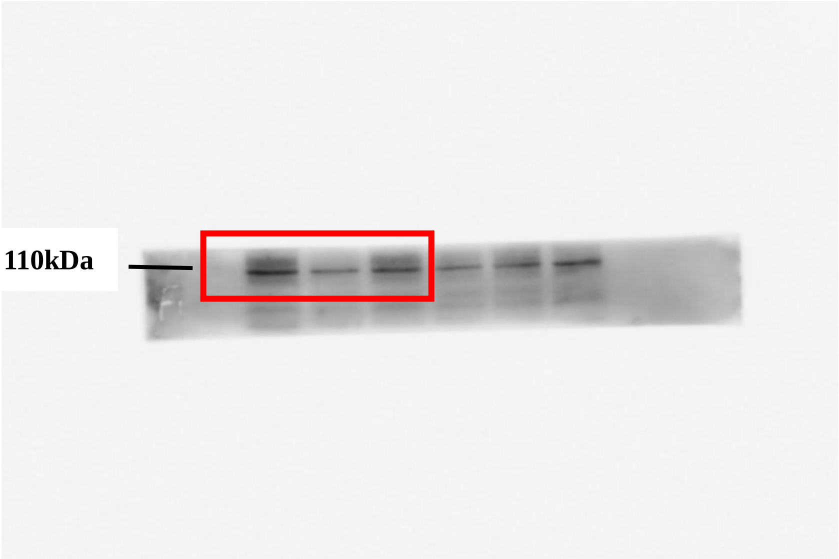

h. p-Akt- Hippocampus

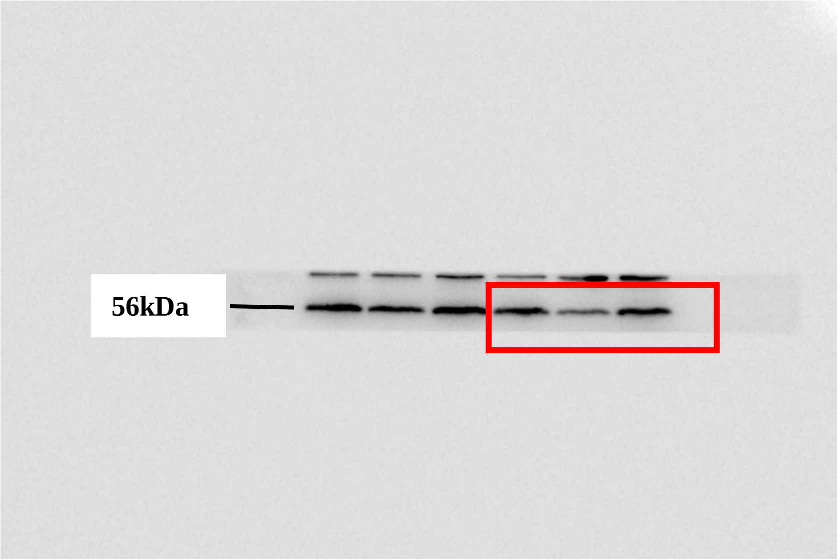

i. Akt- Hippocampus

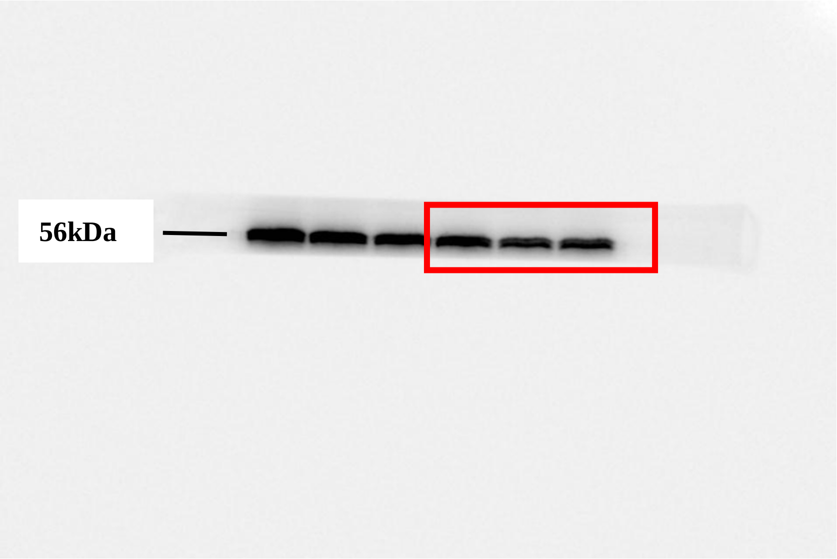

j. p- GSK3β- Hippocampus

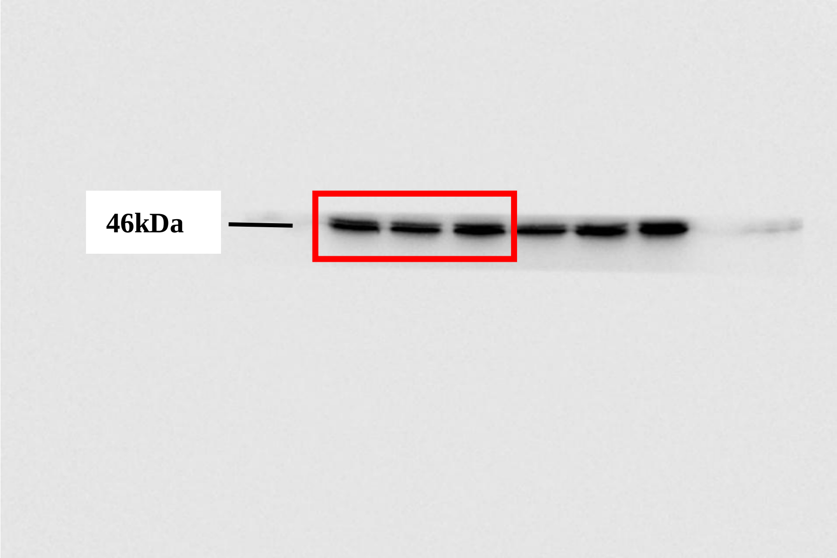

k. GSK3β- Hippocampus

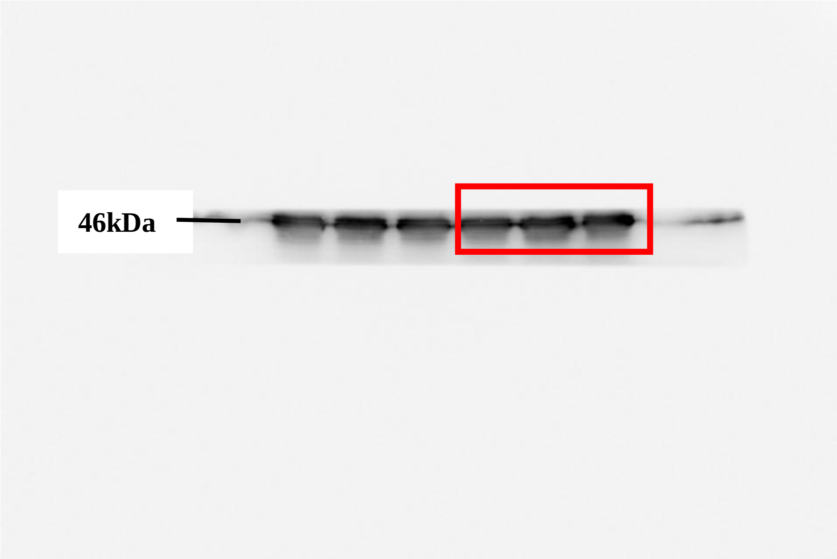

l. actin- Hippocampus

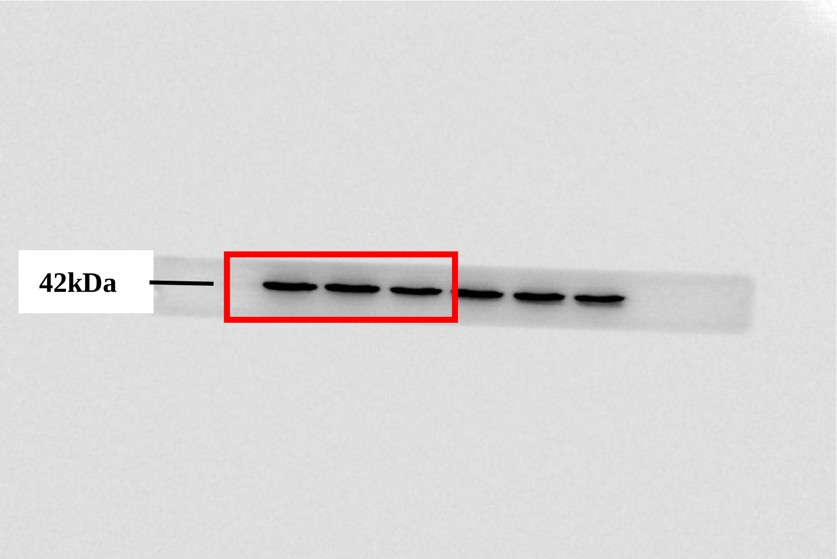

Supplement: Supplementary file 1 — Supplementary Material [file CNS-28-1108-s001.pdf]
